# BioRels’ data infrastructure: a scientific schema and exchange standard to transform and enhance biological data sciences

**DOI:** 10.1093/nar/gkaf254

**Published:** 2025-04-04

**Authors:** Jibo Wang, Amanda Turney, Lauren Murray, Andrew M Craven, Patty Bragger-Wilkinson, Bruno dos Santos, Jaroslav Martasek, Jeremy Desaphy

**Affiliations:** Lilly Genetic Medicines, Eli Lilly and Company, Indianapolis, IN 46285, United States; Research-IDS, Eli Lilly and Company, Indianapolis, IN 46285, United States; Research-IDS, Eli Lilly and Company, Indianapolis, IN 46285, United States; Tech@Lilly, Eli Lilly and Company, Indianapolis, IN 46285, United States; Tech@Lilly, Eli Lilly and Company, Indianapolis, IN 46285, United States; Lilly Genetic Medicines, Eli Lilly and Company, Indianapolis, IN 46285, United States; Tech@Lilly, Eli Lilly and Company, Indianapolis, IN 46285, United States; Lilly Genetic Medicines, Eli Lilly and Company, Indianapolis, IN 46285, United States

## Abstract

Our understanding of biology and medicinal sciences augmented by advances in data structures and algorithms has resulted in proliferation of thousands of open-sourced resources, tools, and websites that are made by the scientific community to access, process, store, and visualize biological data. However, such data have become increasingly complex and heterogeneous, leading to an entangled web of relationships and external identifiers. Despite emergence of infrastructure such as data lakes, the scientists are still responsible for the time consuming and costly exercise to find, extract, clean, prepare, and maintain such data sources while following the FAIR principles. To better understand the complexity, we lay down a representation of the mainstream data ecosystem, describing the natural relationships and concepts found in biology. Built upon it and the fundamental principles of data unicity and atomicity, we introduce BioRels, an automated and standardized data preparation workstream aiming at improving reproducibility and speed for all scientists and handling up to 145 billion data points. BioRels allows complex querying capabilities across several data sources seamlessly and provides an exchange format, BIORJ, to export and import data with all its dependency and metadata. At last, we describe the advantages, limitations, applications, and perspectives of a future approach BioRels-KB to expand future data preparation capabilities.

## Graphical Abstract

**Graphical Abstract:**
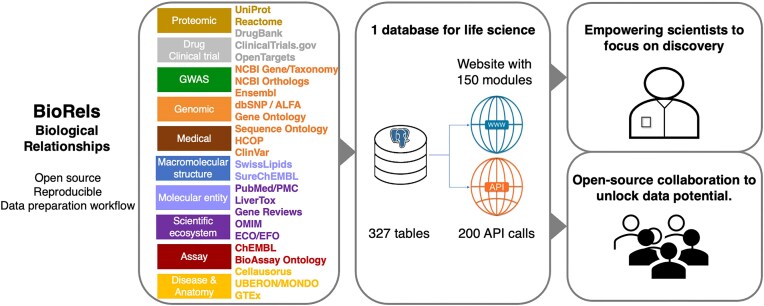

## Introduction

Scientific decisions are fundamentally rooted in data, particularly in the realm of biological sciences. These data encompass a wide array of elements spanning various domains such as proteomics, genomics, genetics, small and large molecules, and clinical trials. Each of these domains comes with its own unique nomenclature and representation, which is not preordained but rather evolves gradually through community efforts. Over time, tools and platforms are developed, and websites are established to facilitate the accessibility of this data. As understanding deepens, consensus sometimes arises, leading to the emergence of key drivers within the scientific community. UniProt [1], ClinGen [2], InterPro [3], and wwPDB [4] are perfect and diverse representations of such drivers for proteins, clinical relevance of variants, protein family, and macromolecular structures, respectively. These institutions or consortia assume the responsibility of establishing standards within their respective domains, serving as the guiding force for others to adhere to as the field advances. But for many domains, no consensus can emerge due to the large variability, competitive interests, and the varying lifecycles of data sources, which often face issues like obsolescence or poor maintenance [5]. Indeed, as the scientific community navigates the intricate landscape of data-driven decision-making in biological sciences, the myriad tools and websites at our disposal offer both opportunities and challenges.

Today, >2236 databases [6], 30 307 tools [7], and 250 ontologies [8] stand ready to aid researchers in their quest for knowledge, providing unprecedented access to data across many scientific domains. However, alongside this abundance arises a set of challenges that must be addressed to fully leverage the potential of these resources. The vast amount of data accessible through the multitude of platforms and websites can lead to a “data swamp”, where valuable information becomes obscured within an ocean of irrelevant, unsecured, low-quality, obsolete, or deeply buried data. This overwhelming influx can trigger a phenomenon known as “choice overload,” making it harder for researchers to identify the best tools or resources for their work. In turn, they may end up spending more time comparing options rather than focusing on their research. Additionally, the entangled and heterogeneous connectivity between various tools and datasets can pose obstacles to seamless integration and analysis.

Navigating the vast expanse of data is akin to swimming in a boundless sea, with various techniques tailored to specific needs [9]. One approach involves a distributed model, constructing a cohesive library of computational tools to seamlessly query and merge data from diverse sources. The Bio* Project [10, 11], spearheaded by the Open bioinformatics foundation, exemplifies global collaboration, streamlining access to bioinformatics tools for the wider community. Alternatively, early endeavors like the Integrated Genome Database (IGD) in 1994 attempted a centralized data warehouse, amalgamating over 12 databases. However, the nascent internet and technology posed challenges, leading to frequent schema changes and the eventual breakdown of the IGD system, as communicated by O. Ritter and Lincoln D. Stein [9]. In contrast, InterMine [12, 13], developed by the Micklem Lab at the University of Cambridge, stands as a contemporary substitute. InterMine processes ∼30 diverse data sources, primarily focusing on genomics, and serves as the foundational infrastructure for 19 websites within the InterMine Registry. Each website offers unique insights tailored to specific organisms, providing a nuanced perspective on the available information.

In addition to the multifaceted challenges facing scientific data management, a range of initiatives that are not necessarily directed towards sciences has emerged to foster greater accessibility, reliability, privacy, and quality. The FAIR principles [14], among others, have had a tremendously positive impact as a framework for enhancing the findability, accessibility, interoperability, and reusability of research data. Orthogonally, the European Union General Data Protection Regulation (https://commission.europa.eu/law/law-topic/data-protection_en, last accessed: 11 March 2025) stands as a pivotal framework for safeguarding Europeans overall privacy and personal rights. Meanwhile, initiatives such as ALCOA+ (https://www.fda.gov/media/119267/download, last accessed: 11 March 2025), Title 21 CFR Part 11 (https://www.ecfr.gov/current/title-21/chapter-I/subchapter-A/part-11 last accessed: 11 March 2025), and of course the International Organization for Standardization (ISO) norms set standards for data quality and management, particularly in industries subject to stringent regulatory oversight.

Yet, one of the most critical concerns and current area of focus is reproducibility, as the reliance on numerous tools, datasets and data sources increases the complexity of replicating scientific findings. On an infrastructural level, initiatives driven by national and international funding bodies have sought to address or anticipate issues of connectivity and reproducibility. The NCBI [15] remains a pioneering force in building comprehensive life sciences infrastructure, with similar initiatives such as EMBL-EBI [16] with the Elixir consortium [17] in Europe, the CNCB-NGDC [18] in China, SIB [19] in Switzerland, or the Global Biodata Coalition. Those initiatives aim at promoting data harmonization and accessibility by improving the inter-connectivity, open-accessibility, and relationships between the different data sources to reduce data duplications and improve scientific quality. Despite the large positive strides made by those, this has led however to the unexpected consequences of creating a data jigsaw, leaving the scientists piecing the resources together, even between those well-established infrastructures, thus increasing the risk for mistakes and heterogenicity in the preparation process. In addition, it is leaving less-supported infrastructures vulnerable to being engulfed in the data swamp. This highlights the ongoing need for innovative solutions throughout the data lifecycle. Indeed, the data preparation stage, involving tasks such as data acquisition, cleaning, and organization, remains a time-consuming bottleneck in scientific data endeavors, underscoring the importance of continued efforts to streamline and optimize data management processes. As of today, while crucial for reproducibility, this aspect remains unaddressed by any existing infrastructure.

In proposing a transformative approach to scientific data management, we advocate for the establishment of a novel infrastructure that shifts the burden of data preparation from individual scientists to the broader research community. This innovative framework aims to enhance reproducibility and streamline research efforts by centralizing data preparation processes within a single, cohesive data warehouse infrastructure. This infrastructure is designed to mirror the logical relationships inherent in scientific concepts such as proteins, genes, small molecules, and variants, thereby facilitating consistency and standardization across datasets. By uniquely defining each scientific concept, this framework fosters homogenization and ensures data unicity. To realize this vision, we will initially outline a partial representation of the drug discovery data ecosystem, elucidating the key considerations informing its development. Subsequently, we will introduce the proof-of-concept of this infrastructure as a data mart, empowering scientists to customize their data sources and organism selections while maintaining full integrity and interdependency with other resources. Through this paradigm shift, researchers can focus their efforts on advancing scientific inquiry, confident in the robustness and accessibility of their data infrastructure.

## Materials and methods

### Drug discovery data landscape

The landscape of drug discovery data has been meticulously crafted through an approach that begins with delineating some of the foundational scientific concepts: proteins, genes, drugs, clinical trials, 3-D structures, disease, anatomy, and small molecules. Subsequently, a thorough manual examination of data sources ensues, elucidating additional scientific principles and delineating the interrelationships among them. To streamline terminology, dependencies are defined as the relationship between a parent concept and its dependent child concept. These dependencies are further categorized into critical and related dependencies. Critical dependencies are pivotal for maintaining the scientific integrity of a given record, such as the association of an organism to a gene record, without which crucial scientific knowledge would be lost. Conversely, related dependencies augment a record, providing supplementary information like external identifiers or publications. Any scientific concept without a critical dependency will be set as root level (L1), while those with dependencies are assigned a level corresponding to the maximum dependency level of their parent concepts plus one.

To build the corresponding database schema, BioRels operates on two fundamental principles: atomicity and unicity. This means that when multiple data sources encompassing the same scientific concept are encountered, they are consolidated based on their identifiers, while retaining unique entries for each. To enhance connectivity, every scientific concept is meticulously deconstructed into its most granular component. For instance, each amino acid within a protein sequence is represented as an individual record, and likewise, each DNA nucleotide within a chromosome is defined distinctly. The complete database schema is available in Supplementary Fig. S1 and is subdivided by the ecosystems.

### Infrastructure

This infrastructure works using an SGE cluster, all the data are stored in a POSIX system and in a PostgreSQL database. The backend infrastructure is executed within a singularity container, which contains all the third-party tools and libraries necessary, allowing to run a set of 160 scripts, mainly written in PHP and Python3.12. BioRels can be adapted to run using other job schedulers or on a single CPU (slower).

The front-end website, written using PHP v7, Datatables toolkit, Reactome’s Fireworks JS widget, jquery, jquery UI, and an Apache server, provides a web interface for scientists. Similarly, the website can be run in a Docker container. The website is divided into modules that can surface the data in different format: Webpage, text, excel, fasta, image, json, 3-D file format (MOL2/PDB), among other formats.

At last, an Application Programming Interface (API) is available both on the website and in the backend infrastructure with >200 queries.

Source code for both the infrastructure and the website are available under GPL3 at https://github.com/EliLillyCo/BioRels.

### BioRels as a data mart—selection data sources

Following the compilation of the container and establishment of the database schema, scientists are prompted to engage in a series of pivotal decisions. First, they are invited to select genome assemblies from NCBI or Ensembl, if applicable, as well as proteomes from UniProt, if desired. Subsequently, they can handpick data sources from a comprehensive pool of 31 options, tailoring the dataset to their specific requirements. Moreover, scientists can choose from a plethora of processing options and additional computational methodologies to suit their analytical needs. For licensed data sources such as OMIM or DrugBank, provision of login credentials is required. The subsequent phase of the process involves an automated examination of critical dependencies to seamlessly integrate parent data sources, ensuring a comprehensive and coherent dataset, without additional requirements from the scientist.

### Computational framework

The data processing workflow is orchestrated by a master script along with a suite of 160 processing scripts to handle the 31 data sources listed in Table 1. Each data source undergoes processing by one or more scripts, depending on its complexity. Initially, a daily check determines if a new release is available for a specific data source. If an update exists, the download process is initiated. Subsequently, the processing scripts come into play, but only after confirming that all parent data sources have been successfully updated first, ensuring data integrity and consistency. In the event of a process failure, downstream scripts remain on hold until resolution. Once the process for a given data source is complete, the data from the previous version is removed from the filesystem and the version of the data is updated in the database in a specific versioning table.

**Table 1.** List of data sources covered in BioRels with their update frequency and licensing

Data type	Data Source	Release frequency	License
Taxonomy	NCBI Taxonomy [92]	Daily	NCBI Policy
Gene	NCBI Gene [15] Ensembl [22]	Daily Bi-Weekly	NCBI Policy CC BY 4.0
Orthologs	NCBI Orthologs [15] HCOP [23]	Daily	NCBI Policy EMBL-EBI Policy
Variant/Mutation	dbSNP [93]	No schedule	NCBI Policy
Small molecule	ChEMBL [48]	No schedule	CC BY-SA 3.0
Clinical variant	Clinvar [55]	Weekly	NCBI Policy
RNA expression	GTEx [56]	Version 8	CC BY-NC-SA 3.0
Protein domain	InterPro [3]	1–3 Months	EMBL-EBI Policy
Drug	DrugBank [68]	Weekly	DrugBank license
Disease	MONDO [57]	Bi-Monthly	CC BY 4.0
Anatomy	UBERON [58]	Bi-Monthly	N/A
Small molecule/Lipid	SwissLipids [49]	Weekly	CC BY 4.0
Small molecule/Patent	SureChEMBL [50]	No schedule	EMBL-EBI Policy
Cell lines	Cellosaurus [59]	No schedule	CC BY 4.0
Disease/Drug/Clinical trials	Open Targets [94]	Bi-Monthly	CC 0
Disease ontology	EFO [95]	Monthly	Apache 2.0
Sequence ontology	SO [24]	No schedule	CC BY-SA 4.0
Evidence ontology	ECO [73]	Monthly	CC0 1.0
BioAssay ontology	BAO [96]	No schedule	CC BY 4.0
Protein information	UniProt [1]	Quarterly	CC BY 4.0
Publication	PubMed [15]	Daily	NCBI Policy
Pathway	Reactome [35]	Bi-monthly	Public domain
Gene ontology	GO [25]	Monthly	CC BY 4.0
Gene/DNA/RNA	RefSeq [97] Ensembl [22]	No schedule	NCBI Policy EMBL-EBI Policy
Clinical Trials	US Clinical Trials	Daily	US CT.gov Terms of use
Disease/Gene information	OMIM [60]	Daily	OMIM License
Gene information	Gene Reviews^®^ [98]	Frequently	Gene Reviews Copyright
Liver toxicity	LiverTox [99]	Frequently	Freely available
PubMed central (PMC)	PMC [15, 74]	Daily	CC Only

EMBL-EBI terms of use: https://www.ebi.ac.uk/about/terms-of-use/; NCBI data policy: https://www.ncbi.nlm.nih.gov/home/about/policies/; Gene Reviews copyright notice and usage: https://www.ncbi.nlm.nih.gov/books/NBK138602/; US clinical Trials: https://www.clinicaltrials.gov/about-site/terms-conditions.

In addition to the incorporation of those data sources, BioRels offers the possibility to perform additional computations. For instance, during each Uniprot update, pairwise protein sequence and domain alignments are generated using a combination of blastp [20] and smith-waterman sequence alignment [21]. After either Uniprot or RefSeq/Ensembl update, transcript to protein translation is performed by using EMBOSS transeq [20]. At last, it also provides the ability to query Pubmed against genes, diseases, drugs, and tissues. A complete description of the method used for each data source is provided in Supplementary Text S1.

## Results

### Map of drug discovery data ecosystem

**Figure 1.**
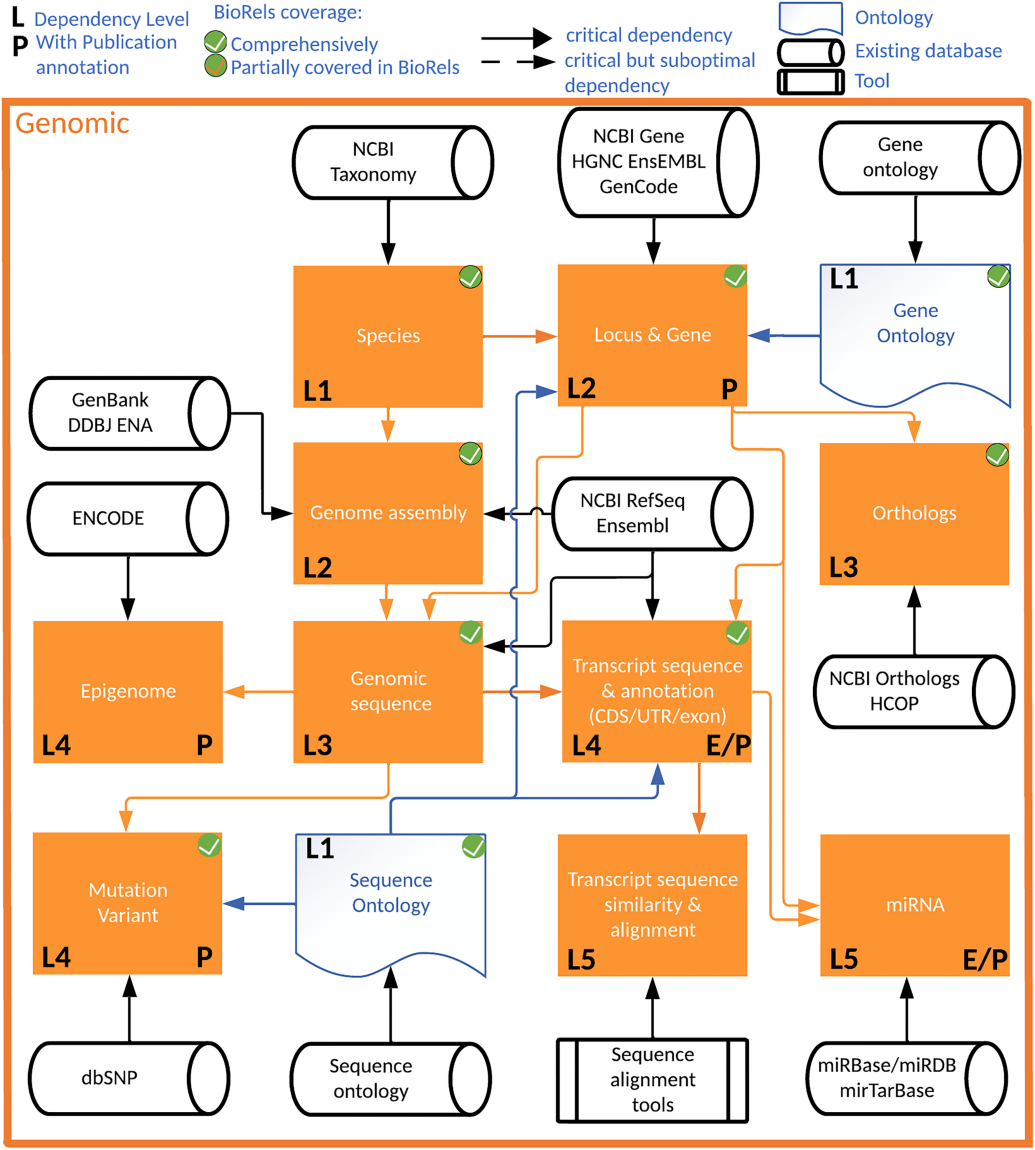
Genomic ecosystem. Public/private resources are represented as rounded black direct data shapes, Tools as rectangular black predefined process, Ontologies as blue documents, and Genomic concept as orange rectangular process. Each arrow describes the directionality of the dependency: From the parent scientific concept to the child scientific concept that depends on it. The L[N] represent the level of dependency depth of a scientific concept, i.e. the minimum number of dependency layer to comprehensively describe this scientific concept. A “P” on the bottom right corner describes a scientific concept which can be associated with publications, while a “E” describes annotated record by the Evidence and Conclusion Ontology.

Establishing a strong data integration infrastructure for drug discovery relies on understanding how different scientific concepts from various data sources are connected. Although mapping the entirety of the drug discovery data ecosystem is beyond the scope, our focus lies on delineating the principal drivers. To streamline visualization, we categorized resources into ten ecosystems, each representing overarching scientific concepts. The *Genomic* ecosystem—presented in Fig. 1—encapsulates organisms, DNA, RNA, and other genomic and genetics constituents. All the other ecosystems are provided in Supplementary Figs S2–S10. Each scientific concept is represented by box colored by the ecosystem it is in. The dependency between two scientific concepts is described by an arrow going from the parent concept to the child concept. For example, a gene (child) record only exists in the organism (parent) it is defined in. The dependency level for a given scientific concept is defined as the maximum number of dependency layers required to get all the necessary information to process that scientific concept. Genomic sequence, i.e. the DNA sequence of each chromosome, would require to first process organisms’ data, as provided by NCBI Taxonomy, followed by the genome assembly of interest and the chromosomal information for this organism, provided by NCBI Gene, Ensembl. Therefore, genomic sequence is the third layer of dependency prior to processing variant, epigenome, and transcript data [15, 22–34]. In addition to the Genomic ecosystem, nine other ecosystems have been defined. Proteomic delves into protein sequences, domains, and annotations [1, 3, 25, 35–46]. Macromolecular structure elucidates structural insights derived from nuclear magnetic resonance (NMR), X-ray, and cryo-EM techniques [4, 47]. A molecular entity delineates diverse molecule types, such as small molecules, antibodies, silencing RNAs (siRNAs), lipid nanoparticles, or a combination thereof [48–54]. *Disease/anatomy* ecosystem elucidates anatomical structures across different organisms and encompasses various diseases and their impacts at different levels [2, 15, 25, 55–67]. The *assay* ecosystem encapsulates the different type of assays: single protein, cellular or *invivo* studies as well as the generated experimental data, synergizing with molecular structure, disease/anatomy, genomic, and proteomic ecosystems [48, 51, 66]. Drugs advancing to clinical stages are detailed in a distinct ecosystem, including clinical outcomes, on-target/off-target effects, and adverse event reports [15, 48, 68, 69] (ATC: https://www.who.int/tools/atc-ddd-toolkit/atc-classification; EU Clinical trials: https://euclinicaltrials.eu/; China Clinical trials: https://www.chictr.org.cn/indexEN.html; Canada MedEffect: https://www.canada.ca/en/health-canada/services/drugs-health-products/medeffect-canada.html; FDA Adverse event reporting system: https://www.fda.gov/drugs/fdas-adverse-event-reporting-system-faers/fda-adverse-event-reporting-system-faers-public-dashboard; last accessed: 11 March 2025). The medical ecosystem would therefore be patient centric, covering an individual ancestry, genome, and medical record [70–72] (ICD-11: https://icdcdn.who.int/icd11referenceguide/en/html/index.html, last accessed: 11 March 2025). All of them are highly connected to the scientific community ecosystem, encompassing scientists, institutions, and the various published documents such as patents, publications, and reports [15, 73, 74]. At last, genome wide association studies is defined with its own ecosystem [75–78] due to its staggering complexity intricately intertwined with many other ecosystems.

### Converting the data ecosystem into an infrastructure

The infrastructure we’ve developed is grounded on three pivotal assumptions, each reflecting the current landscape of technological capabilities and scientific needs. First, it’s predicated on the notion that technological advancements enable the processing of vast quantities of data with unprecedented efficiency. Second, these same advancements facilitate the management of complex data structure and querying of data across diverse scientific domains, effectively flattening complex datasets into accessible formats. Third, it acknowledges that from the perspective of most scientist, the requisite scale of data isn’t as expansive as one might assume. To substantiate these assumptions, we constructed an infrastructure utilizing 31 publicly available data sources, encompassing 35 scientific concepts across 7 distinct ecosystems. It accommodates up to twelve levels of dependencies, nearing the upper limit found in the data ecosystem. Notably, our efforts included comprehensive coverage of genomic and proteomic data for eleven organisms listed in Supplementary Table S1, alongside mRNA/protein translation, protein sequence, and protein domain sequence similarity and alignment. A glimpse at Table 2 reveals the scale of our endeavor, with 145 billion records housed within a single 27-terabyte database. However, the resources required for a typical laboratory can be accommodated with just 1 terabyte of storage, making this level of access feasible for most small organizations.

**Table 2.** Number of records in million and size in gigabytes based on the ecosystem

	**Simplified**		**Complete**	
	Records	Size	Records	Size
Macromolecular Structure	4.3	0.6	4.3	0.6
Assay	6.3	2.6	6.3	2.6
Community	709.0	196.8	19 100.0	6196.8
Disease	1521.0	211.7	4920.8	629.9
Drug	265.4	47.6	265.4	47.6
Genomic	645.6	138.4	64 436.6	9518.3
GWAS	0.0	0.0	0.0	0.0
Molecular Entity	120.7	85.0	120.7	85.0
Other	0.3	30.8	0.3	30.8
Proteomic	2338.8	409.2	56 525.2	10 182.4
Total	5611.3	1122.7	145 379.4	26 694.0

COMPLETE represent the entirety of the data currently processed and managed by BioRels, while SIMPLIFIED does not consider genomic assemblies, variants, translations, and alignments. Please note that Macromolecular structure and GWAS ecosystems have not been processed in this release.

### Querying the infrastructure—tools and applications

**Figure 2.**
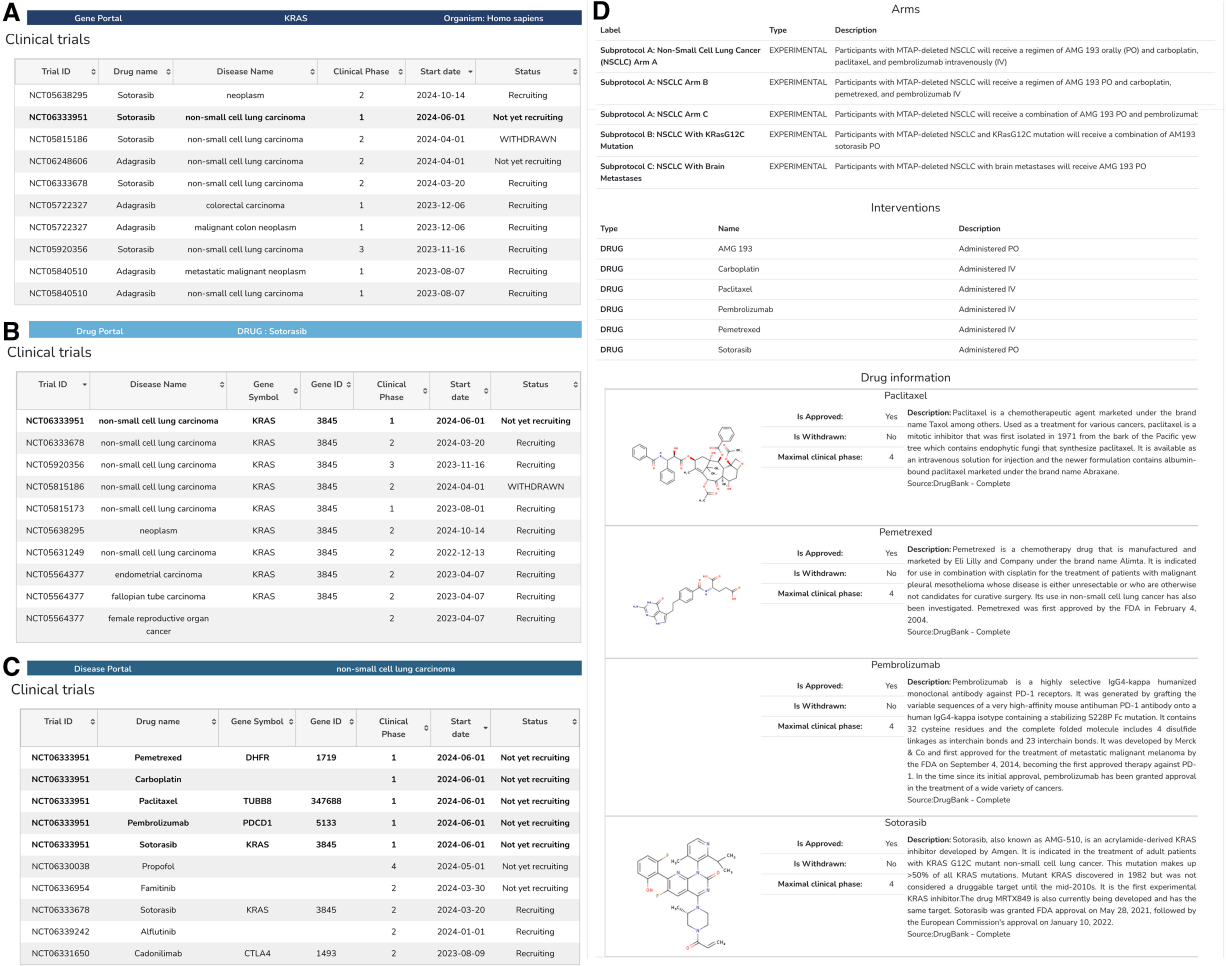
Example of webpage and data integration with Clinical trial. (**A**) list of clinical trials for drugs targeting KRAS. (**B**) List of clinical trials for Sotorasib drug. (**C**) List of clinical trials reported for nonsmall cell lung carcinoma disease. Panels (A), (B), and (C) highlight the clinical trial NCT0633591 in bold. (**D**) Arm and intervention description for NCT0633591 clinical trial. Provides additional information like the drug structure and textual description from DrugBank and OpenTargets and links to the drug webpage.

BioRels stands as a pioneering platform, offering a versatile website that unveils data through various lenses, segmented into the previously described ecosystems termed portals. Each portal comprises reusable modules tailored to showcase data pertinent to one or multiple scientific concepts. Clinical trial information such as NCT0633951 should be retrievable when searching by KRAS gene target (Fig. 2A), Sotorasib drug (Fig. 2B), or nonsmall cell lung carcinoma condition (Fig. 2C). Each of them should link to the clinical trial page showing not only the information provided by the clinical trial itself but also metadata such as drug structure or description (Fig. 2D).

Moreover, every module possesses the capability to seamlessly integrate with others, as well as facilitating data conversion into multiple formats like JSON, webpage, or FASTA, effectively operating as an API with minimal overhead. If a website isn’t an option, scientists can query the backend API providing >200 queries spanning across the ecosystems. While the process of loading each nucleotide of a genome individually may seem time-consuming and overly meticulous at first glance, its querying capabilities prove exceptionally efficient. Retrieving 100 000 nucleotides from any chromosome takes merely 2 s, while compiling all variants associated with a specific gene requires <5 s.

**Figure 3.**
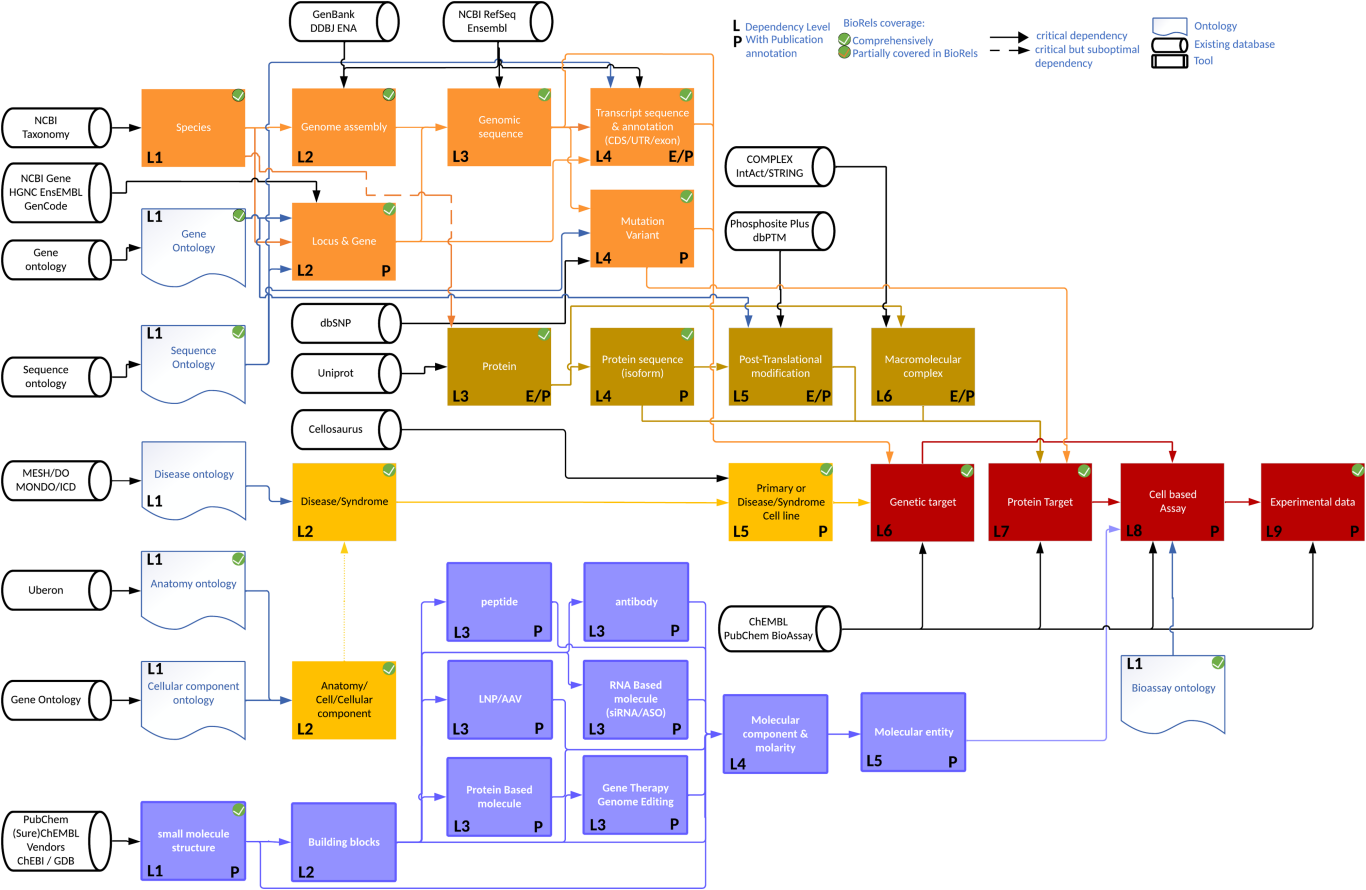
Representation of BIORJ dependency map for the export of a cell based assay and its experimental data (red boxes on right hand side). The dependencies levels are layered vertically for better representation. Rounded black direct data shape: Potential public/private data source. Light blue documents: Ontology. Rectangles: scientific concepts. Dark yellow: Proteomic scientific concept. Melrose: Molecular entity scientific concept. Orange: Genomic scientific concept. Yellow: Disease/anatomy concept. Red: assay concept. Each arrow describes the directionality of the dependency: From the parent scientific concept to the child scientific concept that depends on it. The L[N] represent the level of dependency depth of a scientific concept, i.e. the minimum number of dependency layer to comprehensively describe this scientific concept. A “P” on the bottom right corner describes a scientific concept which can be associated with publications, while a “E” describes annotated record by the Evidence and Conclusion Ontology.

However, the true innovation lies in our export/import feature, which not only captures the desired data but, owing to its inherent integration of dependencies, extracts metadata and all necessary parent data as well, maintaining the data integrity from top to bottom. For example, the export of a cell-based assay and its experimental data (Fig. 3), respectively defined with a dependency level of 8 and 9, can be done seamlessly and would include the data export of up to 30 different scientific concepts—when applicable—in a simple command. This feature converts any record into a simple JSON data structure called BIORJ, which can be further linearized into a simple text file. The BIORJ format can streamlines data sharing between BioRels infrastructures, potentially fostering effortless collaboration, and even sharing complete datasets.

### Scientific applications

**Figure 4.**
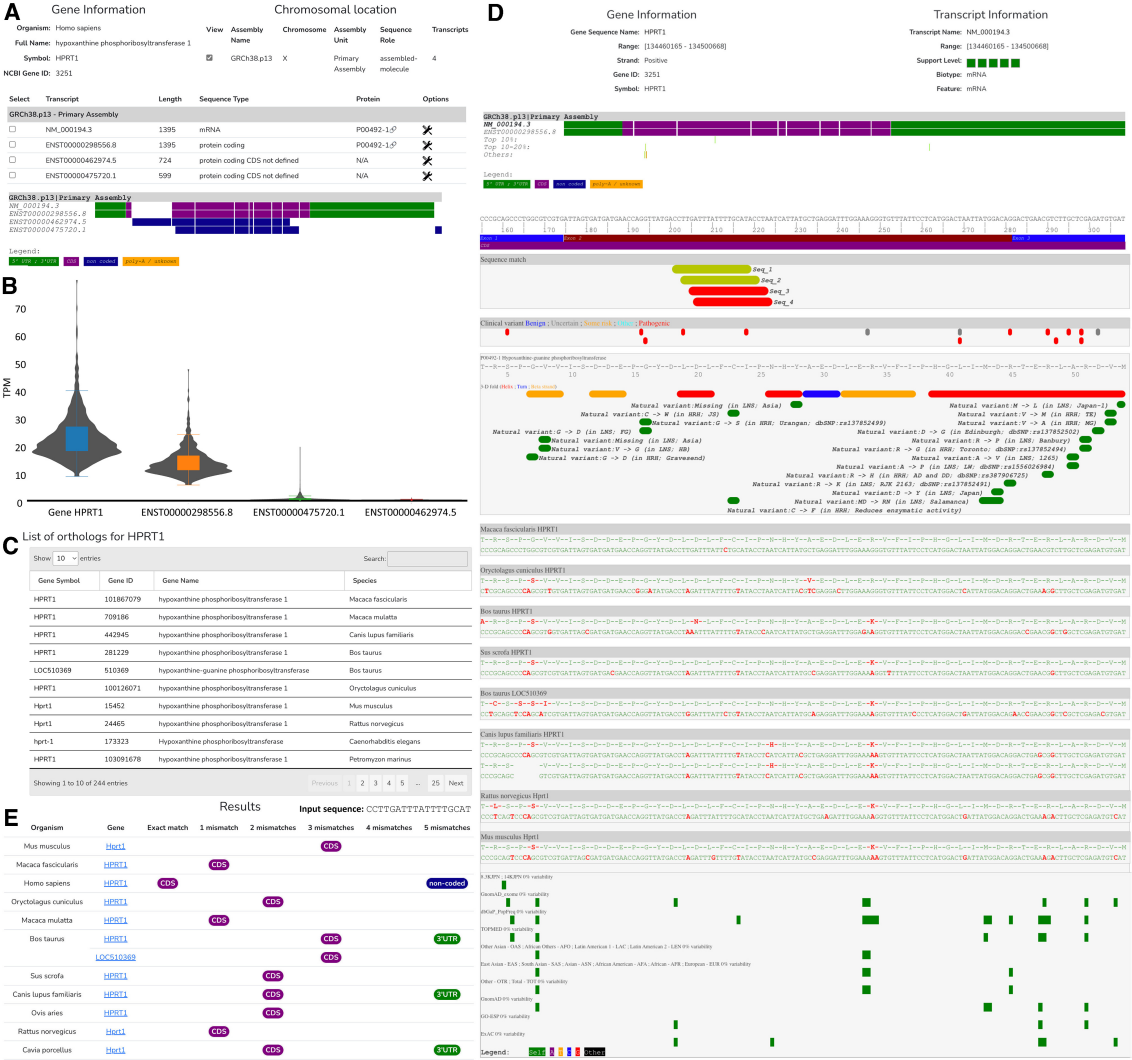
Suite of webpages supporting ASO and siRNA drug discovery from early target evaluation to experimental results with HRPT1 as example. (**A**) List of reported HPRT1 transcripts with their annotations (NCBI Gene, RefSeq, and Ensembl). (**B**) RNA expression data in frontal cortex tissue for HPRT1 gene and transcripts (GTEX). (**C**) List of HPRT1 orthologous genes (NCBI Orthologs, HCOP). (**D**) Mapping of randomly generated siRNA sequences color-coded by their activity data against HPRT1 NM_000194.3 transcript. Top: transcript/gene information and global view of the alignment. Mapping of reported clinical variant (ClinVar), protein translation and annotation (UniProt), orthologous sequence alignment [different or identical amino-acid or nucleotide] and DNA variant frequency information, aggregated by frequency profile across different studies. (**E**) Match of a siRNA sequence against all HPTR1 transcripts across different organisms, providing the number of mismatch and localization.

**Figure 5.**
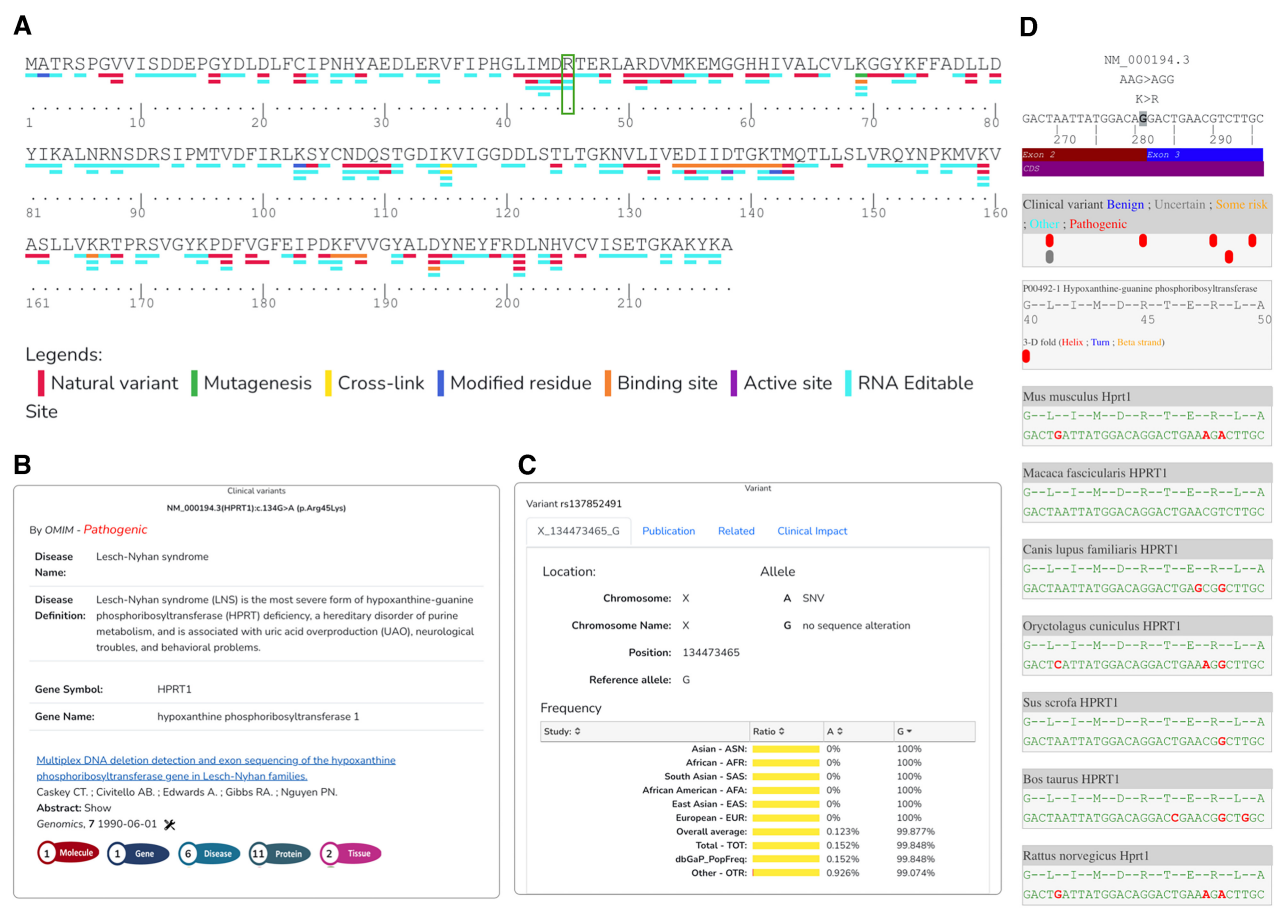
Evaluation of suitable RNA editable sites for Human HPRT1 protein sequence. (**A**) Each colored rectangle under an amino-acid represent a UniProt annotation or a RNA editable site (cyan). The selected amino-acid R45 is highlighted by the vertical rectangle. Generated web report providing information about the RNA editable site HPRT1 K > R45 from ClinVar (**B**) and dbSNP (**C**). (**D**) Representation of the mRNA targeted region with cross-reactivity information based on protein sequence alignment between HPRT1 human and HPRT1 orthologs as well as mRNA to protein translation. Highlighted in red are the mismatches between the Human mRNA and protein sequence versus orthologs mRNA and protein sequences, respectively.

Today, BioRels is an integral component of Lilly Institute for Genetic Medicines, as it plays a pivotal role in sequence selection and analysis for antisense oligonucleotides, siRNA (Fig. 4) as well as mature RNA (mRNA) target evaluation for RNA editing (Fig. 5). For any given Human target, BioRels allows a scientist to easily examine via the website the known transcripts from both NCBI and Ensembl (Fig. 4A), compare them and choose the most expressed ones based on RNA expression levels (Fig. 4B). Next, a proprietary algorithm tiles the transcript(s) to generate the initial pool of siRNAs and provide an assessment for each of them. This includes DNA variability within the target region, on-target profile and cross-reactivity based on orthology (Fig. 4C), off-target risks in Human and other organisms and miRNA off-target risk. After experimental testing, we can map the potent siRNA sequences onto the target transcript (Fig. 4D) to verify their location, on/off-target profiles, and cross-reactivity to other organisms (Fig. 4E). Those analysis, requiring the use of dbSNP, ALFA, NCBI gene, Ensembl, HCOP, NCBI orthologs, and RefSeq are easily made possible by the fact that the data are centralized and properly connected.

A similar approach has been applied to address the emerging modality of RNA editing, which enables the modification of RNA by changing an adenosine to an inosine using an Editing Oligonucleotide. This modification is interpreted during translation as a guanine (G), offering potential therapeutic benefits. Notably, G > A missense and nonsense mutations account for 20% (21 134/103 235) of pathogenic single-nucleotide variants (SNVs) reported in ClinVar (https://www.ncbi.nlm.nih.gov/clinvar/, accessed: 11 March 2025). This makes identifying all adenosines that could potentially be edited particularly valuable for assessing their therapeutic potential. BioRels already houses many of the necessary data sources for such an analysis, including gene information, genome and transcript data, variant and allele frequencies, as well as protein sequence and mRNA-to-protein translation information. Leveraging these data, we developed a process to evaluate all adenosines reported in human protein-coding transcripts. Given that pathogenic mutations are typically low-frequency variants, we expanded our search to include all adenosines reported in dbSNP within these transcripts. This process then assesses whether editing a given adenosine to guanine would result in an alteration of the protein sequence or its translation. Our analysis identified 261 358 832 DNA positions mapped to Human protein-coding transcripts with a reported adenosine, either in the transcript or as a variant. Of these, 39 012 377 positions would lead to an amino acid modification, while 226 630 positions could prevent premature termination of translation. BioRels facilitates the visualization of these potential RNA editing sites at the protein sequence level, cross-referencing them with UniProt annotations. Scientists can then access BioRels website to look for the different annotations provided by UniProt on a protein sequence as well as the identified RNA editable sites (Fig. 5A). Next, they have the possibility to select an editable site to review information provided by ClinVar, the publications associated with the record (Fig. 5B) and dbSNP (Fig. 5C). Thanks to the precomputed protein sequence alignment and mRNA/protein translation, the website also offers a full insight on the targeted mRNA transcript sequence with ClinVar, Uniprot protein sequence and annotation as well as protein and mRNA mismatch with orthologous genes for cross-reactivity evaluation across other organisms (Fig. 5D). This integrated approach thus offers a powerful tool for evaluating RNA editing opportunities and their therapeutic implications.

## Discussion

Technology is advancing at an incredible pace, and the data generated from it continues to grow even faster. To push further the boundaries of our biological knowledge, scientists are increasingly required to become excellent data steward in how they find, retrieve, prepare, maintain, generate, and share data, with the FAIR principles used as guidance. Despite being crucial for scientific reproducibility and progress, this process is inherently complex and variable, and has been historically challenging. Consequently, evaluating the efficacy of data preparation infrastructure can be difficult. One approach to gauge its potential benefits is through assessing the return on investment of existing infrastructure. The EMBL’s European Bioinformatics Institute, which manages >150 data resources and tools, reported in its 2021 Executive Summary Report that the wider value of their resources is equivalent to 23 to 102 times the total estimated costs. In addition, the de-duplication of research efforts could be worth 6 billion pounds per year (https://www.embl.org/documents/wp-content/uploads/2021/10/EMBL-EBI-impact-report-summary-2021.pdf; last accessed: 11 March 2025). Similarly, a 2017 report on the economic impact of using RCSB-PDB resources was estimated at 800 times greater than its direct operating cost (https://cdn.rcsb.org/rcsb-pdb/general_information/about_pdb/Economic%20Impacts%20of%20the%20PDB.pdf; last accessed: 11 March 2025). Thus, enhancing data access and preparation processes stands poised to significantly benefit the scientific community and ultimately the people they serve. We aim at addressing such challenge by proposing a new infrastructure that would be positioned between the data resources and the scientists. In helping the scientists to “shop” for the data sources they need and offering a standard framework for data preparation, we intend in reducing the data preparation burden and Human cost, improving the accessibility, the integrity and ultimately the reproducibility of the data.

To successfully build such infrastructure, understanding the intricacies of how the data flows and the relationships between the different data components is paramount, leading to the creation of a biological and drug discovery map. With >2000 biological databases reported, it is impractical if not unfeasible to describe in its totality the drug discovery data ecosystem, which is very vast and complex. Therefore, we have chosen to focus on outlining the heavily cited resources and didn’t cover bacterial, fungi, viruses’, and toxicological databases. To simplify its readability, we broke it down into 10 ecosystems, each covering a specific biology or drug discovery domain, and further broken down into scientific concepts with commonly used vocabulary: Protein, Gene, Molecule, and Clinical trial. Each scientific concept is then connected to other concepts when it is either a part of, a consequence of, or is associated with it. For instance, a clinical trial can assess the safety and efficacy of a drug candidate for a given condition/disease. As such, drug and disease information are critical dependencies of a clinical trial. Thus, a clinical trial database is a child of the parents’ disease and drug. The critical dependencies defined in this map doesn’t however perfectly match the reality as trade-offs are inevitable due to experimental constraints. A prototypical example resides in how UniProt annotates its protein entries. In an ideal world, a protein should be annotated by the gene that codes for its sequence. However, the identification and confirmation of the coding gene can come later after the discovery of the protein. As such, UniProt annotates protein records by organisms and provide gene annotations.

This analysis has revealed the importance of ontologies, being with NCBI Taxonomy the foundational layer of the ecosystem and underlining the critical importance of standardized nomenclature in data quality and reusability. Furthermore, ∼90 resources have been highlighted as primary catalysts for the proliferation of biological and drug discovery data, underscoring their paramount importance in advancing scientific understanding and innovation. After defining all the critical dependencies, we were surprised to see the relatively shallow depth of these relationships, with 76 out of 120 scientific concepts accessible within five layers of dependencies or less. To cover the bulk of drug discovery data, our analysis has revealed that thirteen levels of dependencies suffice to cover almost all the main scientific concepts. This suggests that building a robust data preparation infrastructure is not only achievable but also within reach, offering the potential to significantly streamline drug discovery processes in the future.

### BioRels—a data warehouse/mart

To prove the feasibility of such concept, we have developed BioRels, a biological relationship infrastructure. With the capacity to ingest data from 31 data sources in real-time, BioRels covers 35 out of 120 scientific concepts, spanning across 7 data ecosystems and accommodating up to 9 levels of dependencies within the presented data landscape. However, understanding how BioRels fits within the current infrastructure paradigm is important. In today’s world of big data, four types of infrastructure have been proposed: a data lake, a data warehouse, a data mart, and knowledge graphs.

A data lake serves as an expansive storage facility where data of any format—be it images, documents, PDFs, free text, structured, or unstructured—can reside at various curation stages. This freedom allows data to flow into the lake without immediate processing requirements, enabling data scientists to query it with exceptional flexibility. However, this flexibility comes with trade-offs, including the lack of structure and architecture, necessitating scientists to possess expert domain knowledge regarding the type, quality, format, and associated metadata of the data they seek [79]. In contrast, a data warehouse presents a more structured infrastructure with predefined data schemas. While this imposes upfront costs for data preparation and loading, it also limits immediate access to the data and imposes constraints and filters based on the schema. Yet, these limitations are offset by the advantages of clean, homogenized, and interconnected data. Alternatively, a Data Mart can be viewed as a subset of a data warehouse, tailored to address specific business needs. Each of these infrastructures has their own set of benefits and challenges, extensively discussed in numerous publications [79, 80]. Additionally, an emerging alternative infrastructure that has been gaining significant traction over the last decade is the knowledge graph, particularly in the medical domain [81, 82]. Its ease of use, coupled with the ability to quickly load vast and heterogeneous datasets, makes it an invaluable tool for inferring connections and deriving insights. Despite these advantages, knowledge graphs are increasingly recognized for their lack of an enforced data structure, which limits their suitability for efforts aimed at reintroducing structure into heterogeneous data. This challenge highlights the need for more robust frameworks that can balance flexibility with organization, especially in data-intensive fields like healthcare.

The current system most resembling BioRels is InterMine, developed by the Micklem lab in 2002. Tailored to present genomic information for a set of organisms, InterMine allows scientists to build their own data model during configuration, which the system will adapt to unify and load data from multiple sources. It then merges information covering the same entity, operating as a model-driven data warehouse that usually loads data at build time, with some specific exceptions. InterMine is now the foundation for >19 instances, including FlyMine [83] and TargetMine [84]. In comparison, BioRels embodies elements of both a data warehouse and a data mart, offering a predefined data schema that grants scientists the flexibility to choose which data sources, genomes, and proteomes to incorporate. Although InterMine provides greater freedom in how data are framed, BioRels predefined data schema provides a stable and standardized foundation to build upon and share data between systems. As such, either system can be used depending on the scientific needs.

#### Benefits and limitations

The inherent limitations of a data warehouse infrastructure are rooted in its fundamental concept. First, scientists encounter the challenge of upfront costs associated with ingesting data before it can be utilized effectively. This initial investment can be significant both in terms of storage and computation, depending on the number of data sources and their coverage. However, it can be seen in BioRels as a one-time fee, with minimal Human cost thanks to the automation, and little extra cost for updates. Additionally, the requirement to ingest data into a predefined schema poses constraints on flexibility and adaptability. As the database schema expands to accommodate more data sources, any modifications to the schema, particularly for foundational resources at the L1 or L2 levels in the data landscape, can potentially trigger a cascading downstream effect. This can be mitigated in part by separating the critical information of a given data source from its annotation. For instance, a gene would be defined by its locus, identifier(s), symbol, and organism, while synonyms, alias, textual description can be separated in other tables. Such principle of narrowing down a scientific concept to its critical information would minimize the risk of cascading effect, while retaining the ability to expand around it as much as necessary. Over time, as the scientific community gains a deeper understanding of the data and its relationships, the schema will gradually stabilize, improving its long-term adaptability. As the infrastructure expand, the number of tools and libraries required can also become a bottleneck. One potential solution would be to evolve the containerization into a container mart, where each data source provides their list of dependencies, thus allowing to build tailored containers based on the scientist needs.

This infrastructure offers also numerous advantages, primarily through the enforcement of a unique definition for each scientific concept, thereby streamlining and homogenizing information. However, managing multiple data sources concurrently has historically been in a bioinformatic challenge in maintaining consistency. BioRels’ unique design is the first infrastructure that fully incorporates and enforces critical dependencies in its design, in which the system's architecture dictates that child data sources (for instance a gene) are updated only upon successful updating of parent data sources (such as the taxon), guaranteeing the utmost accuracy during processing. Furthermore, BioRels preserves relationships even when parent data sources are updated, maintaining data consistency even if child sources become inactive or infrequently updated. This enables older data sources to maintain their relevance, as their relationships can be kept up to date, reducing the risk of connectivity loss seen in data lakes. Additionally, if a parent record becomes obsolete or is removed, BioRels automatically deletes all related child records in cascade. While this could pose a challenge for reproducibility, the issue can be mitigated using BioRels’ BIORJ export/import capability, which allows users to take a complete snapshot of a dataset with all its dependencies at any given time, ensuring that the data and its relationships remain reusable.

### Perspectives on the current state of the public data ecosystem

#### A unified data community

The multitude of databases and tools crafted by scientists over recent decades has yielded a diverse landscape characterized by varying levels of quality, accessibility, and more importantly redundancy. As scientific knowledge evolves, the community continually hones specific concepts until they attain a level of maturity conducive to establishing a comprehensive definition. Transforming this definition into a sturdy foundation becomes imperative for progress. Yet, complexities arise when economic considerations, national policies, and competitive forces intersect, resulting in a lot of scientific concepts presented in the data landscape to be described by multiple data sources. Converging towards a consensus—even an imperfect one—becomes imperative in navigating this complexity. A successful example of such consensus is InterPro [3]. Through the integration of thirteen member databases, InterPro offers a unified classification system for protein families and domains, fostering homogeneity and ease of use. This streamlined approach must be applied to all well-established scientific concepts, even as underlying processing rules evolve. For instance, a more unified collaboration between RefSeq and Ensembl, extending beyond the scope of the MANE initiative, could bring significant benefits to the scientific community [85, 86]. Genomes and transcriptomes serve as foundational elements for many downstream research concepts, and the differences between these resources often lead to cascading effects. Initiatives inspired by projects like InterPro could reduce the number of identifiers, minimizing data redundancy, complexity, and the potential for errors. Such a shift would ultimately lower the time, cost, and effort required from researchers, while fostering the creation of dynamic, adaptable resources that can continue to evolve and improve over time.

#### Open source licenses

Even with advancements in data sharing, licensing remains a significant challenge. The complexity, variability of licensing terms, as well as their accessibility—sometimes very well hidden—can impede the seamless utilization of scientific data. While the diversity of open-source licenses presents challenges—many of which were not designed with scientific contexts in mind—protecting patient privacy, ensuring proper credit for researchers, and supporting appropriate use of data remain fundamental. To mitigate these concerns, a tailored legal framework for scientific licenses is needed, one that differentiates between academic, medical, personal, and commercial uses of data and tools, as well as research activities within industry settings. This framework should distinguish between commercial uses that directly drive profit, such as developing new treatments or medical products for sale, and research activities within industry settings, such as academic-industry collaborations or post-doctoral work in a corporate environment, which contribute to scientific discovery but are not primarily focused on driving the business. Such a system would foster innovation, ensure the proper use of scientific data, and maintain compliance with legal and privacy standards, while strengthening trust and collaboration between academic institutions and industry to drive collective progress. To address this, it is essential for the scientific community to take an active role in establishing a transparent licensing framework, grounded in clear and standardized principles that prioritize ethical use, legal compliance, and data/tool stewardship. In turn, we envision a system in BioRels where users can more clearly activate the use of licenses (approved within their organization) based on the type of organization and use case. This would allow BioRels to quickly verify whether a data source can be used, either in its entirety or partially, by automating the assignment of the correct license and ensuring compliance, streamlining data access decisions, and monitoring potential changes in license terms. With BioRels as an open source framework, the scientific community itself would play a key role in ensuring that licensing terms are respected and implemented, alleviating some of the weight and concerns around this issue and promoting a shared responsibility for data stewardship.

#### Reproducibility

Reproducibility, which relies on having identical data and tools to achieve consistent results, remains a significant challenge. While the goal of full reproducibility is commendable, the vast volume of data, the variability of tool versions, and the preparation processes make it an ongoing, complex task. One potential way to address this complexity is through centralization, where a broad user base works within a unified infrastructure, potentially reducing some of the variability. However, even within centralized systems, many data sources still lack proper versioning mechanisms. For example, NCBI Gene provide a history file to map outdated gene records to their current counterparts, but they do not offer the full set of data—such as synonyms or complete names—which limits the ability to trace specific versions over time and complicates the task of reproducing exact processes. Many others only provide monthly or quarterly historical versions.

We recognize the technological and resource challenges involved in achieving full versioning, especially for dynamic resources like NCBI Taxonomy and Gene, which are frequently updated. BioRels faces similar challenges on both the technological and data fronts. For instance, when a taxon is removed from NCBI Taxonomy or a disease record becomes obsolete, there’s a dilemma: should related data from other sources be removed to preserve data integrity and reflect the latest scientific consensus, or should the data be retained to support consistency with the FAIR principles, even if it risks accumulating outdated, irrelevant or inaccurate information due to a retracted publication? This balancing act—between maintaining data accuracy and ensuring reproducibility, especially at that scale—illustrates the complexities scientists face as they navigate the rapidly evolving landscape of scientific data.

Centralization could help address this by consolidating datasets into a unified infrastructure, ensuring that, while not identical, a given dataset remains more consistent and aligned across various sources. This approach allows for greater accuracy in reproducing results, even as some data may evolve over time. By operating within a centralized system, discrepancies between versions can be minimized, making reproducibility more achievable without compromising the overall integrity of the data.

To address this, BioRels adopts a flexible approach: it applies scientific data integrity constraints, assuming that if a user provides the same version of input data and the same BioRels version, the resulting BioRels database will be identical. Additionally, BioRels offers users the ability to export a list of data sources along with their versions, or to export datasets as Biorj files for easier sharing and reusability. One can then shares the complete dataset to be loaded in a BioRels database, even with obsolete records and with minimal overhead to reproduce the results of a new method for example. Obsolete records would remain in the database until a refresh is triggered.

### Future development

**Figure 6.**
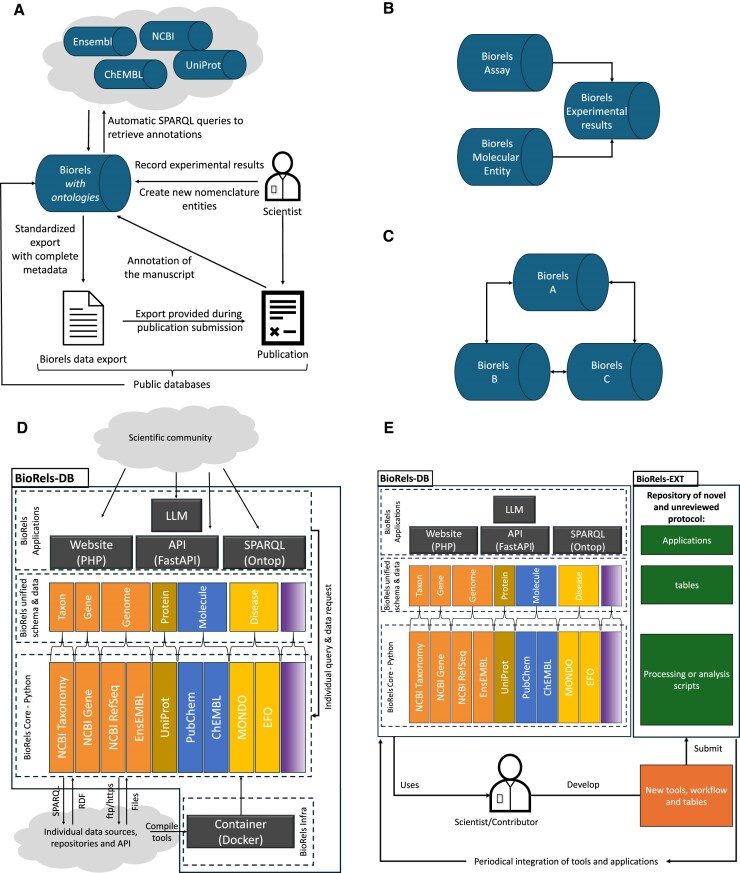
Description of potential avenues for BioRels. (**A**) BioRels as a scientific support infrastructure. (**B**) Data infrastructure as multiple data silo, each handling a different ecosystem and sharing data using BIORJ files to a third instance that is dependent on their data. (**C**) Multiple data infrastructure sharing their data to one another. (**D**) Overview of BioRels future infrastructure. (**E**) Overview of the process for scientist to use and expand BioRels. BioRels-EXT can serve as a repository for scientific protocols, which over time can be ingested in the core BioRELS-DB infrastructure.

Currently, BioRels is designed to support the large-scale use of data resources and aid in the development of new resources that integrate multiple scientific concepts. As such, it is not well-suited for smaller laboratories or teams that lack access to high-performance computing systems. However, the foundational principles of BioRels can be extended to create a more flexible, on-demand infrastructure. By leveraging semantic web technologies and SPARQL [87] along with BioRels’ dependency management logic, this approach offers a scalable solution that could accommodate a wider range of users. We envision a platform where scientists directly input and document their findings, experiments, and images into the database, adhering to BioRels’ data schema standards. SPARQL queries would ensure the completeness of the data, and its dependencies based on the data ecosystem (Fig. 6A). With ontologies fully integrated into the database, a scientist’s manuscript could be preannotated with relevant scientific concepts as well as the experimental data, streamlining the process. Ultimately, this system would make newly generated data, its dependencies, and manuscript annotations readily available during the publication process thanks to a BIORJ file, enhancing efficiency, collaboration and ingestion by public resources. The completeness of the data, which would include information from dependent data sources, would also have an indirect, yet crucial impact of supporting public resources by tracking their impact [88].

With the current state of BioRels arises a multitude of possibilities in leveraging its capabilities. In a production environment in large organizations, the necessity, if not the criticality, of segregating the data architecture into discrete sub-components becomes evident. We can imagine a simple scenario—Fig. 6B where two BioRels-DB instances are dedicated to assay registration and molecular entity registration. Thanks to its standardized data schema, both can share their information using BIORJ data exchange to a third instance storing experimental results. BIORJ format not only encompasses the pertinent data but also ensures comprehensive data integrity by including all relevant parent data, easily allowing to harness the system’s flexibility and scalability. Alternatively, each data source can have their own BioRels instance, easily communicating and sharing data in real time with one another and to scientists around the world Fig. 6C. Scientists could therefore query each data source for a specific data point and get all the associated metadata with it.

We envision the creation of BioRelsKB (Biological Resource Knowledge Base), a community-driven infrastructure designed to advance biological and drug discovery data science. BioRelsKB could not only provide a stable core but also allow for the development of new tools and workflows. Drawing inspiration from systems like UniProtKB, BioRels could differentiate between curated, quality-assured data (BioRels-DB, Fig. 6D) and user-contributed, unreviewed extensions (BioRels-EXT, Fig. 6E). BioRels-DB could offer high-quality, expert-curated data sources, scripts, and database tables, maintained by the community to serve as a solid foundation for scientific research. Meanwhile, BioRels-EXT could host user-contributed tools, protocols, and extensions submitted by scientists. These contributions could undergo periodic community review and once validated, could be integrated into the system, ensuring the continual evolution of the platform.

Built on a robust schema defined by experts from leading data providers, BioRelsDB could offer a stable foundation for managing, processing, and visualizing biological data. The core schema could be reviewed periodically to ensure it adapts to evolving scientific needs while warranting stability. Core data processing would be handled in Python, with each data provider responsible for maintaining their own data processing scripts (bulk data and SPARQL queries) to upload data into a central PostgreSQL database.

To support the diverse needs of the scientific community, BioRelsDB could enable flexible customization through Docker containers via an infrastructure called BioRelsDock. These containers could automatically compile the necessary packages and tools for selected data sources, ensuring that researchers can quickly and easily set up their working environments without the need for manual configuration. This approach provides scalability and reproducibility, allowing scientists to focus on their research rather than the technical setup. BioRelsDB could offer four core applications: A customizable website that provides both simple and complex visualizations via modular components, a standard API for general data access, a SPARQL API for more specific data queries. To enable natural language queries, the integration of Retrieval-Augmented Generation methods (e.g. Gorilla [89]) that queries the API could help mitigate inaccuracies commonly seen in global Large Language Models like ChatGPT [90]. These components could streamline workflows, support collaboration, and ensure that scientists have immediate access to high-quality data, tools and visualization. The core infrastructure of BioRelsDB could remain stable, with modifications only made by the core data providers and BioRels team, ensuring consistency and reliability across the platform.

Scientists could be able to tailor BioRelsDB to their specific project needs by selecting the data sources they require. Once the data sources are chosen, the system could automatically trigger the build of a customized container with all necessary packages and tools. This flexibility ensures that researchers can quickly adapt BioRels to their unique workflows, enabling faster and more efficient data analysis. The applications could enable scientists to quickly build their own website to share their results, without having to reinvent the wheel and while benefiting from what the community has the best to offer.

## Conclusion

BioRelsKB has the potential to evolve beyond a mere collection of individual tools and data sources into a collaborative, community-driven infrastructure that can catalyze scientific discovery at an unparalleled scale. The database schema’s inherent flexibility, designed to reflect biological relationships, not only provides a strong foundation for innovation, homogeneity and open science organizations [91], but also enables continuous adaptation to emerging scientific concepts and methodologies. By bringing together diverse data providers, domain experts, and cutting-edge technologies, we can create a platform that seamlessly integrates high-quality data while fostering growth, adaptability, and collaboration. Just as each component of BioRelsKB brings its unique strengths, the collective efforts of the community ensure that together we achieve more than the sum of our parts. We have demonstrated its pivotal role in drug discovery, particularly in genetic medicine, where BioRelsKB enables scientists to visualize complex, heterogeneous data in an accessible way, supporting all siRNA and ASO preclinical projects. By working together, we can build a more efficient, flexible, and impactful foundation for biological and drug discovery, accelerating innovation and driving progress in ways no single entity could accomplish alone.

## Supplementary Material

gkaf254_Supplemental_Files

## Data Availability

The repository underlying this article is available in https://github.com/EliLillyCo/BioRels. A snapshot is available at https://dx.doi.org/10.5281/zenodo.13921143. A capsule is available in Code Ocean at https://doi.org/10.24433/CO.2933957.v1

## Notes

Present address: Amazon, Detroit, MI 48226, United States

Present address: Genomics Research Center, Abbvie, North Chicago, IL 60064, United States

## References

[1] The UniProt Consortium UniProt: the Universal protein knowledgebase in 2025. Nucleic Acids Res. 2024; 53:D609–17. 10.1093/nar/gkae1010. PMC11701636 39552041

[2] Rehm HL, Berg JS, Brooks LD et al. ClinGen—the Clinical genome resource. N Engl J Med. 2015; 372:2235–42. 10.1056/NEJMsr1406261. 26014595 PMC4474187

[3] Blum M, Andreeva A, Florentino Laise C et al. InterPro: the protein sequence classification resource in 2025. Nucleic Acids Res. 2024; 53:D444–56. 10.1093/nar/gkae1082. PMC11701551 39565202

[4] wwPDB consortium Protein Data Bank: the single global archive for 3D macromolecular structure data. Nucleic Acids Res. 2018; 47:D520–8. 10.1093/nar/gky949. PMC6324056 30357364

[5] Wren JD, Bateman A Databases, data tombs and dust in the wind. Bioinformatics. 2008; 24:2127–8. 10.1093/bioinformatics/btn464. 18819940

[6] Rigden DJ, Fernández XM The 2025 Nucleic Acids Research database issue and the online molecular biology database collection. Nucleic Acids Res. 2025; 53:D1–9. 10.1093/nar/gkae1220. 39658041 PMC11701706

[7] Ison J, Rapacki K, Ménager H et al. Tools and data services registry: a community effort to document bioinformatics resources. Nucleic Acids Res. 2016; 44:D38–47. 10.1093/nar/gkv1116. 26538599 PMC4702812

[8] Côté R, Reisinger F, Martens L et al. The ontology Lookup service: bigger and better. Nucleic Acids Res. 2010; 38:W155–60. 10.1093/nar/gkq331. 20460452 PMC2896109

[9] Stein LD Integrating biological databases. Nat Rev Genet. 2003; 4:337–45. 10.1038/nrg1065. 12728276

[10] Cock PJA, Antao T, Chang JT et al. Biopython: freely available Python tools for computational molecular biology and bioinformatics. Bioinformatics. 2009; 25:1422–3. 10.1093/bioinformatics/btp163. 19304878 PMC2682512

[11] Stein L Creating a bioinformatics nation. Nature. 2002; 417:119–20. 10.1038/417119a. 12000935

[12] Kalderimis A, Lyne R, Butano D et al. InterMine: extensive web services for modern biology. Nucleic Acids Res. 2014; 42:W468–72. 10.1093/nar/gku301. 24753429 PMC4086141

[13] Smith RN, Aleksic J, Butano D et al. InterMine: a flexible data warehouse system for the integration and analysis of heterogeneous biological data. Bioinformatics. 2012; 28:3163–5. 10.1093/bioinformatics/bts577. 23023984 PMC3516146

[14] Wilkinson MD, Dumontier M, Aalbersberg IJ et al. The FAIR guiding principles for scientific data management and stewardship. Sci Data. 2016; 3:160018 10.1038/sdata.2016.18. 26978244 PMC4792175

[15] Sayers Eric W, Beck J, Bolton Evan E et al. Database resources of the National Center for Biotechnology Information in 2025. Nucleic Acids Res. 2025; 53:D20–9. 10.1093/nar/gkae979. 39526373 PMC11701734

[16] Thakur M, Brooksbank C, Finn Robert D et al. EMBL’s European Bioinformatics Institute (EMBL-EBI) in 2024. Nucleic Acids Res. 2025; 53:D10–9. 10.1093/nar/gkae1089. 39607697 PMC11701561

[17] Harrow J, Drysdale R, Smith A et al. ELIXIR: providing a sustainable infrastructure for life science data at European scale. Bioinformatics. 2021; 37:2506–11. 10.1093/bioinformatics/btab481. 34175941 PMC8388016

[18] CNCB-NGDC Members and Partners Database Resources of the National Genomics Data Center, China National Center for Bioinformation in 2025. Nucleic Acids Res. 2024; 53:D30–44. 10.1093/nar/gkae978. PMC11701749 39530327

[19] SIB Swiss Institute of Bioinformatics RDF Group Members The SIB Swiss Institute of Bioinformatics Semantic Web of data. Nucleic Acids Res. 2023; 52:D44–51. 10.1093/nar/gkad902. PMC10767860 37878411

[20] Rice P, Longden I, Bleasby A EMBOSS: the European Molecular Biology Open Software Suite. Trends Genet. 2000; 16:276–7. 10.1016/S0168-9525(00)02024-2. 10827456

[21] Smith TF, Waterman MS Identification of common molecular subsequences. J Mol Biol. 1981; 147:195–7. 10.1016/0022-2836(81)90087-5. 7265238

[22] Dyer SC, Austine-Orimoloye O, Azov AG et al. Ensembl 2025. Nucleic Acids Res. 2025; 53:D948–57. 10.1093/nar/gkae1071. 39656687 PMC11701638

[23] Yates B, Gray KA, Jones TEM et al. Updates to HCOP: the HGNC comparison of orthology predictions tool. Brief Bioinform. 2021; 22:bbab155 10.1093/bib/bbab155. 33959747 PMC8574622

[24] Eilbeck K, Lewis SE, Mungall CJ et al. The Sequence ontology: a tool for the unification of genome annotations. Genome Biol. 2005; 6:R44 10.1186/gb-2005-6-5-r44. 15892872 PMC1175956

[25] The Gene Ontology Consortium The Gene Ontology Knowledgebase in 2023. Genetics. 2023; 224:iyad031 10.1093/genetics/iyad031. 36866529 PMC10158837

[26] Seal RL, Braschi B, Gray K et al. Genenames.Org: the HGNC resources in 2023. Nucleic Acids Res. 2023; 51:D1003–9. 10.1093/nar/gkac888. 36243972 PMC9825485

[27] Mudge Jonathan M, Carbonell-Sala S, Diekhans M et al. GENCODE 2025: reference gene annotation for human and mouse. Nucleic Acids Res. 2025; 53:D966–75. 10.1093/nar/gkae1078. 39565199 PMC11701607

[28] Sayers EW, Cavanaugh M, Frisse L et al. GenBank 2025 update. Nucleic Acids Res. 2025; 53:D56–61. 10.1093/nar/gkae1114. 39558184 PMC11701615

[29] Kodama Y, Ara T, Fukuda A et al. DDBJ update in 2024: the DDBJ Group Cloud service for sharing pre-publication data. Nucleic Acids Res. 2024; 53:D45–8. 10.1093/nar/gkae882. PMC11701645 39380489

[30] O’Cathail C, Ahamed A, Burgin J et al. The European Nucleotide Archive in 2024. Nucleic Acids Res. 2024; 53:D49–55. 10.1093/nar/gkae975. PMC11701661 39558171

[31] Abascal F, Acosta R, Addleman NJ et al. Expanded encyclopaedias of DNA elements in the human and mouse genomes. Nature. 2020; 583:699–710. 10.1038/s41586-020-2493-4. 32728249 PMC7410828

[32] Kozomara A, Birgaoanu M, Griffiths-Jones S miRBase: from microRNA sequences to function. Nucleic Acids Res. 2019; 47:D155–62. 10.1093/nar/gky1141. 30423142 PMC6323917

[33] Chen Y, Wang X miRDB: an online database for prediction of functional microRNA targets. Nucleic Acids Res. 2020; 48:D127–31. 10.1093/nar/gkz757. 31504780 PMC6943051

[34] Cui S, Yu S, Huang H-Y et al. miRTarBase 2025: updates to the collection of experimentally validated microRNA–target interactions. Nucleic Acids Res. 2025; 53:D147–56. 10.1093/nar/gkae1072. 39578692 PMC11701613

[35] Milacic M, Beavers D, Conley P et al. The Reactome Pathway Knowledgebase 2024. Nucleic Acids Res. 2024; 52:D672–8. 10.1093/nar/gkad1025. 37941124 PMC10767911

[36] Harding SD, Armstrong JF, Faccenda E et al. The IUPHAR/BPS guide to PHARMACOLOGY in 2024. Nucleic Acids Res. 2024; 52:D1438–49. 10.1093/nar/gkad944. 37897341 PMC10767925

[37] del Toro N, Shrivastava A, Ragueneau E et al. The IntAct database: efficient access to fine-grained molecular interaction data. Nucleic Acids Res. 2022; 50:D648–53. 10.1093/nar/gkab1006. 34761267 PMC8728211

[38] Balu S, Huget S, Medina Reyes JJ et al. Complex portal 2025: predicted human complexes and enhanced visualisation tools for the comparison of orthologous and paralogous complexes. Nucleic Acids Res. 2025; 53:D644–50. 10.1093/nar/gkae1085. 39558156 PMC11701666

[39] Szklarczyk D, Kirsch R, Koutrouli M et al. The STRING database in 2023: protein-protein association networks and functional enrichment analyses for any sequenced genome of interest. Nucleic Acids Res. 2023; 51:D638–46. 10.1093/nar/gkac1000. 36370105 PMC9825434

[40] Kanehisa M, Furumichi M, Sato Y et al. KEGG for taxonomy-based analysis of pathways and genomes. Nucleic Acids Res. 2023; 51:D587–92. 10.1093/nar/gkac963. 36300620 PMC9825424

[41] Bairoch A The ENZYME database in 2000. Nucleic Acids Res. 2000; 28:304–5. 10.1093/nar/28.1.304. 10592255 PMC102465

[42] Hornbeck PV, Kornhauser JM, Latham V et al. 15 years of PhosphoSitePlus®: integrating post-translationally modified sites, disease variants and isoforms. Nucleic Acids Res. 2019; 47:D433–41. 10.1093/nar/gky1159. 30445427 PMC6324072

[43] Li Z, Li S, Luo M et al. dbPTM in 2022: an updated database for exploring regulatory networks and functional associations of protein post-translational modifications. Nucleic Acids Res. 2022; 50:D471–9. 10.1093/nar/gkab1017. 34788852 PMC8728263

[44] Perez-Riverol Y, Bandla C, Kundu Deepti J et al. The PRIDE database at 20 years: 2025 update. Nucleic Acids Res. 2025; 53:D543–53. 10.1093/nar/gkae1011. 39494541 PMC11701690

[45] Deutsch EW, Bandeira N, Sharma V et al. The ProteomeXchange consortium in 2020: enabling ‘big data’ approaches in proteomics. Nucleic Acids Res. 2019; 48:D1145–52. 10.1093/nar/gkz984. PMC7145525 31686107

[46] Suzek BE, Wang Y, Huang H et al. UniRef clusters: a comprehensive and scalable alternative for improving sequence similarity searches. Bioinformatics. 2015; 31:926–32. 10.1093/bioinformatics/btu739. 25398609 PMC4375400

[47] Abramson J, Adler J, Dunger J et al. Accurate structure prediction of biomolecular interactions with AlphaFold 3. Nature. 2024; 630:493–500. 10.1038/s41586-024-07487-w. 38718835 PMC11168924

[48] Zdrazil B, Felix E, Hunter F et al. The ChEMBL Database in 2023: a drug discovery platform spanning multiple bioactivity data types and time periods. Nucleic Acids Res. 2024; 52:D1180–92. 10.1093/nar/gkad1004. 37933841 PMC10767899

[49] Aimo L, Liechti R, Hyka-Nouspikel N et al. The SwissLipids knowledgebase for lipid biology. Bioinformatics. 2015; 31:2860–6. 10.1093/bioinformatics/btv285. 25943471 PMC4547616

[50] Papadatos G, Davies M, Dedman N et al. SureChEMBL: a large-scale, chemically annotated patent document database. Nucleic Acids Res. 2016; 44:D1220–8. 10.1093/nar/gkv1253. 26582922 PMC4702887

[51] Kim S, Chen J, Cheng T et al. PubChem 2025 update. Nucleic Acids Res. 2025; 53:D1516–25. 10.1093/nar/gkae1059. 39558165 PMC11701573

[52] Hastings J, Owen G, Dekker A et al. ChEBI in 2016: improved services and an expanding collection of metabolites. Nucleic Acids Res. 2016; 44:D1214–9. 10.1093/nar/gkv1031. 26467479 PMC4702775

[53] Ruddigkeit L, van Deursen R, Blum LC et al. Enumeration of 166 billion organic small molecules in the Chemical Universe Database GDB-17. J Chem Inf Model. 2012; 52:2864–75. 10.1021/ci300415d. 23088335

[54] Wishart DS, Guo A, Oler E et al. HMDB 5.0: the Human Metabolome Database for 2022. Nucleic Acids Res. 2022; 50:D622–31. 10.1093/nar/gkab1062. 34986597 PMC8728138

[55] Landrum Melissa J, Chitipiralla S, Kaur K et al. ClinVar: updates to support classifications of both germline and somatic variants. Nucleic Acids Res. 2025; 53:D1313–21. 10.1093/nar/gkae1090. 39578691 PMC11701624

[56] Lonsdale J, Thomas J, Salvatore M et al. The Genotype-Tissue Expression (GTEx) project. Nat Genet. 2013; 45:580–5. 10.1038/ng.2653. 23715323 PMC4010069

[57] Vasilevsky NA, Matentzoglu NA, Toro S et al. Mondo: unifying diseases for the world, by the world. bioRxiv3 May 2022, preprint: not peer reviewed 10.1101/2022.04.13.22273750.

[58] Mungall CJ, Torniai C, Gkoutos GV et al. Uberon, an integrative multi-species anatomy ontology. Genome Biol. 2012; 13:R5 10.1186/gb-2012-13-1-r5. 22293552 PMC3334586

[59] Bairoch A The Cellosaurus, a cell-line knowledge resource. J Biomol Tech. 2018; 29:25–38. 10.7171/jbt.18-2902-002. 29805321 PMC5945021

[60] Amberger JS, Bocchini CA, Scott AF et al. OMIM.Org: leveraging knowledge across phenotype–gene relationships. Nucleic Acids Res. 2019; 47:D1038–43. 10.1093/nar/gky1151. 30445645 PMC6323937

[61] Schriml LM, Mitraka E, Munro J et al. Human Disease Ontology 2018 update: classification, content and workflow expansion. Nucleic Acids Res. 2019; 47:D955–62. 10.1093/nar/gky1032. 30407550 PMC6323977

[62] Weinreich SS, Mangon R, Sikkens JJ et al. [Orphanet: a European database for rare diseases]. Ned Tijdschr Geneeskd. 2008; 152:518–9. 18389888

[63] Smith JR, Hayman GT, Wang SJ et al. The Year of the Rat: the Rat Genome Database at 20: a multi-species knowledgebase and analysis platform. Nucleic Acids Res. 2020; 48:D731–42. 10.1093/nar/gkz1041. 31713623 PMC7145519

[64] Blake JA, Baldarelli R, Kadin JA et al. Mouse Genome Database (MGD): knowledgebase for mouse-human comparative biology. Nucleic Acids Res. 2021; 49:D981–7. 10.1093/nar/gkaa1083. 33231642 PMC7779030

[65] Uhlen M, Oksvold P, Fagerberg L et al. Towards a knowledge-based Human Protein Atlas. Nat Biotechnol. 2010; 28:1248–50. 10.1038/nbt1210-1248. 21139605

[66] Clough E, Barrett T, Wilhite SE et al. NCBI GEO: archive for gene expression and epigenomics data sets: 23-year update. Nucleic Acids Res. 2024; 52:D138–44. 10.1093/nar/gkad965. 37933855 PMC10767856

[67] Moreno P, Fexova S, George N et al. Expression Atlas update: gene and protein expression in multiple species. Nucleic Acids Res. 2022; 50:D129–40. 10.1093/nar/gkab1030. 34850121 PMC8728300

[68] Knox C, Wilson M, Klinger Christen M et al. DrugBank 6.0: the DrugBank Knowledgebase for 2024. Nucleic Acids Res. 2024; 52:D1265–75. 10.1093/nar/gkad976. 37953279 PMC10767804

[69] Kuhn M, Letunic I, Jensen LJ et al. The SIDER database of drugs and side effects. Nucleic Acids Res. 2016; 44:D1075–9. 10.1093/nar/gkv1075. 26481350 PMC4702794

[70] Gargano MA, Matentzoglu N, Coleman B et al. The Human Phenotype Ontology in 2024: phenotypes around the world. Nucleic Acids Res. 2024; 52:D1333–46. 10.1093/nar/gkad1005. 37953324 PMC10767975

[71] Brown EG, Wood L, Wood S The medical dictionary for regulatory activities (MedDRA). Drug Saf. 1999; 20:109–17. 10.2165/00002018-199920020-00002. 10082069

[72] Rubinstein WS, Maglott DR, Lee JM et al. The NIH genetic testing registry: a new, centralized database of genetic tests to enable access to comprehensive information and improve transparency. Nucleic Acids Res. 2013; 41:D925–35. 10.1093/nar/gks1173. 23193275 PMC3531155

[73] Nadendla S, Jackson R, Munro J et al. ECO: the evidence and conclusion ontology, an update for 2022. Nucleic Acids Res. 2022; 50:D1515–21. 10.1093/nar/gkab1025. 34986598 PMC8728134

[74] The Europe PMC Consortium Europe PMC: a full-text literature database for the life sciences and platform for innovation. Nucleic Acids Res. 2015; 43:D1042–8. 10.1093/nar/gku1061. 25378340 PMC4383902

[75] Sudlow C, Gallacher J, Allen N et al. UK biobank: an open access resource for identifying the causes of a wide range of complex diseases of middle and old age. PLoS Med. 2015; 12:e1001779 10.1371/journal.pmed.1001779. 25826379 PMC4380465

[76] The All of us research Program investigators. The “all of us” research program. N Engl J Med. 2019; 381:668–76. 10.1056/NEJMsr1809937. 31412182 PMC8291101

[77] Kurki MI, Karjalainen J, Palta P et al. FinnGen provides genetic insights from a well-phenotyped isolated population. Nature. 2023; 613:508–18. 10.1038/s41586-022-05473-8. 36653562 PMC9849126

[78] Cerezo M, Sollis E, Ji Y et al. The NHGRI-EBI GWAS Catalog: standards for reusability, sustainability and diversity. Nucleic Acids Res. 2025; 53:D998–D1005. 10.1093/nar/gkae1070. 39530240 PMC11701593

[79] Chen Z Observations and expectations on recent developments of data lakes. Procedia Computer Science. 2022; 214:405–11. 10.1016/j.procs.2022.11.192.

[80] Che H, Duan Y On the logical design of a prototypical data Lake system for biological resources. Front Bioeng Biotechnol. 2020; 8:1–15. 10.3389/fbioe.2020.553904. 33117777 PMC7552915

[81] Mazein I, Rougny A, Mazein A et al. Graph databases in systems biology: a systematic review. Brief Bioinform. 2024; 25:bbae561 10.1093/bib/bbae561. 39565895 PMC11578065

[82] Unni DR, Moxon SAT, Bada M et al. Biolink Model: a universal schema for knowledge graphs in clinical, biomedical, and translational science. Clin Transl Sci. 2022; 15:1848–55. 10.1111/cts.13302. 36125173 PMC9372416

[83] Lyne R, Smith R, Rutherford K et al. FlyMine: an integrated database for Drosophila and Anopheles genomics. Genome Biol. 2007; 8:R129 10.1186/gb-2007-8-7-r129. 17615057 PMC2323218

[84] Chen YA, Tripathi LP, Fujiwara T et al. The TargetMine Data Warehouse: enhancement and updates. Front Genet. 2019; 10:934 10.3389/fgene.2019.00934. 31649722 PMC6794636

[85] Morales J, McMahon AC, Loveland J et al. The value of primary transcripts to the clinical and non-clinical genomics community: survey results and roadmap for improvements. Mol Genet Genomic Med. 2021; 9:e1786. 34435752 10.1002/mgg3.1786 PMC8683622

[86] Morales J, Pujar S, Loveland JE et al. A joint NCBI and EMBL-EBI transcript set for clinical genomics and research. Nature. 2022; 604:310–5. 10.1038/s41586-022-04558-8. 35388217 PMC9007741

[87] Galgonek J, Vondrášek J A comparison of approaches to accessing existing biological and chemical relational databases via SPARQL. J Cheminform. 2023; 15:61 10.1186/s13321-023-00729-5. 37340506 PMC10280967

[88] Ross KE, Bastian FB, Buys M et al. Perspectives on tracking data reuse across biodata resources. Bioinform Adv. 2024; 4:vbae057 10.1093/bioadv/vbae057. 38721398 PMC11076920

[89] Patil SG, Zhang T, Wang X et al. Gorilla: large language model connected with massive APIs. arXiv24 May 2023, preprint: not peer reviewed 10.48550/arXiv.2305.15334.

[90] Sallam M ChatGPT utility in Healthcare education, research, and practice: systematic review on the promising perspectives and valid concerns. Healthcare (Basel). 2023; 11:887 10.3390/healthcare11060887. 36981544 PMC10048148

[91] Edfeldt K, Edwards AM, Engkvist O et al. A data science roadmap for open science organizations engaged in early-stage drug discovery. Nat Commun. 2024; 15:5640 10.1038/s41467-024-49777-x. 38965235 PMC11224410

[92] Cox E, Tsuchiya Mirian TN, Ciufo S et al. NCBI Taxonomy: enhanced access via NCBI datasets. Nucleic Acids Res. 2025; 53:D1711–5. 10.1093/nar/gkae967. 39470745 PMC11701650

[93] Phan L, Zhang H, Wang Q et al. The evolution of dbSNP: 25 years of impact in genomic research. Nucleic Acids Res. 2025; 53:D925–31. 10.1093/nar/gkae977. 39530225 PMC11701571

[94] Buniello A, Suveges D, Cruz-Castillo C et al. Open Targets Platform: facilitating therapeutic hypotheses building in drug discovery. Nucleic Acids Res. 2025; 53:D1467–75. 10.1093/nar/gkae1128. 39657122 PMC11701534

[95] Malone J, Holloway E, Adamusiak T et al. Modeling sample variables with an experimental factor ontology. Bioinformatics. 2010; 26:1112–8. 10.1093/bioinformatics/btq099. 20200009 PMC2853691

[96] Abeyruwan S, Vempati UD, Küçük-McGinty H et al. Evolving BioAssay ontology (BAO): modularization, integration and applications. J Biomed Semant. 2014; 5:S5 10.1186/2041-1480-5-S1-S5. PMC4108877 25093074

[97] Goldfarb T, Kodali Vamsi K, Pujar S et al. NCBI RefSeq: reference sequence standards through 25 years of curation and annotation. Nucleic Acids Res. 2025; 53:D243–57. 10.1093/nar/gkae1038. 39526381 PMC11701664

[98] Adam MP, Feldman J, Mirzaa GM et al. University of Washington, Seattle, copyright © 1993-2025, University of Washington, Seattle. GeneReviews is a registered trademark of the University of Washington, Seattle. 1993; All rights reserved., Seattle (WA).

[99] LiverTox: Clinical and Research Information on Drug-Induced Liver Injury. 2012; Bethesda (MD) National Institute of Diabetes and Digestive and Kidney Diseases. 31643176

