# Supplementary material for: BioRels’ data infrastructure: a scientific schema and exchange standard to transform and enhance biological data sciences
Source: Nucleic Acids Res. 2025 Apr 4;53(6):gkaf254. doi: 10.1093/nar/gkaf254 (PMC11969666; doi:10.1093/nar/gkaf254)
Supplement: gkaf254_Supplemental_Files [file gkaf254_supplemental_files.zip › BioRels_Supplementary_Document.docx]

Supplementary Figure 1 – BioRels database schema – in separate PDF file

| Organism | Assembly | Assembly Name |  |
| --- | --- | --- | --- |
| Mus musculus | GCF_000001635.27 | GRCm39 |  |
| Macaca fascicularis | GCF_012559485.2 | MFA1912RKSv2 |  |
|  | GCA_011100615.1 | Macaca_fascicularis_6.0 |  |
| Homo sapiens | GCF_000001405.39 | GRCh38.p13 |  |
| Oryctolagus cuniculus | GCF_000003625.3 | OryCun2.0 |  |
|  | GCF_009806435.1 | UM_NZW_1.0 |  |
| Macaca mulatta | GCF_003339765.1 | Mmul_10 |  |
| Cavia porcellus | GCF_000151735.1 | Cavpor3.0 |  |
| Rattus norvegicus | GCF_015227675.2 | mRatBN7.2 |  |
| Ovis aries | GCF_016772045.1 | ARS-UI_Ramb_v2.0 |  |
| Canis lupus familiaris | GCF_000002285.5 | Dog10K_Boxer_Tasha |  |
| Sus scrofa | GCF_000003025.6 | Sscrofa11.1 |  |
| Bos taurus | GCF_002263795.2 | ARS-UCD1.3 |  |

Supplementary Table 1 – List of genome assemblies ingested in BioRels for eleven organisms, with their assembly identifier, assembly name.


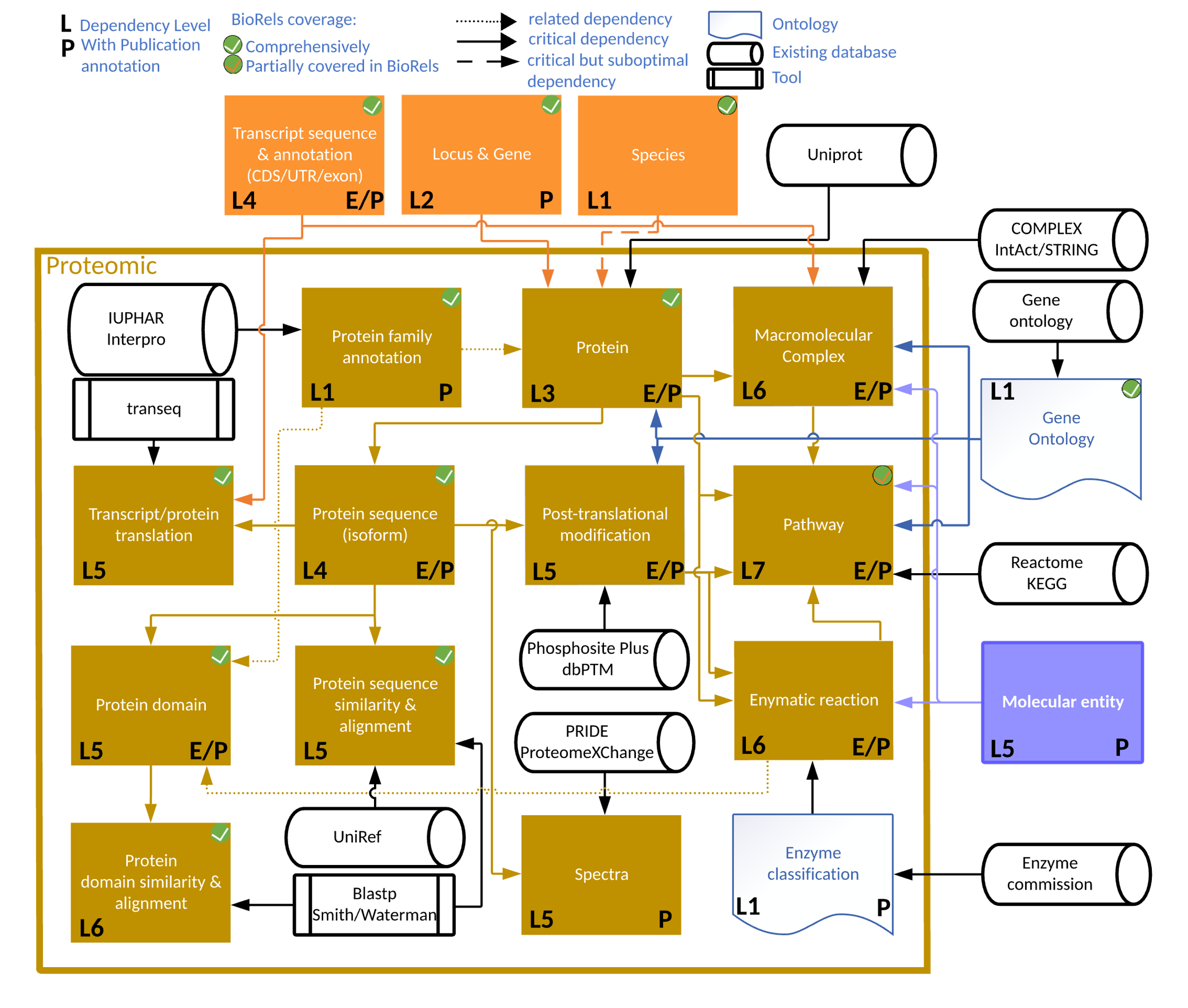


Supplementary Figure 2 Proteomic ecosystem. Rounded Black: Potential public/private data source. Rectangular black box: tools. Light blue shape: Ontology. Dark yellow: Proteomic scientific concept. Melrose: Molecular entity scientific concept. Orange: Genomic scientific concept. Each arrow describes the directionality of the dependency: From the parent scientific concept to the child scientific concept that depends on it. The L[N] represent the level of dependency depth of a scientific concept, i.e. the minimum number of dependency layer to comprehensively describe this scientific concept. A “P” on the bottom right corner describes a scientific concept which can be associated to publications, while a “E” describes annotated record by the Evidence and Conclusion Ontology.


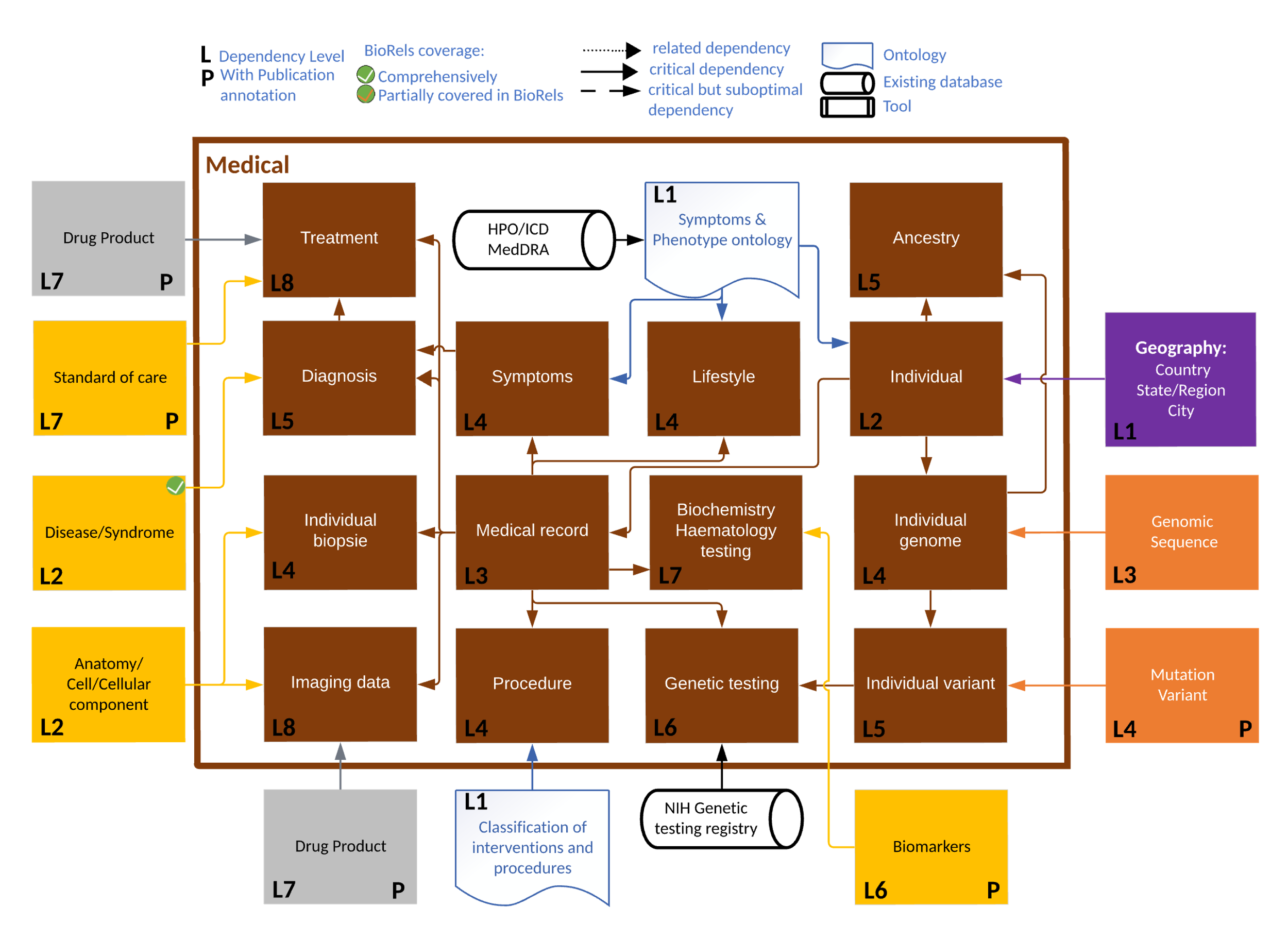


Supplementary Figure 3 Medical ecosystem- Rounded Black: Potential public/private data source. Rectangular black box: tools. Light blue shape: Ontology. Dark yellow: Proteomic scientific concept. Melrose: Molecular entity scientific concept. Orange: Genomic scientific concept. Yellow: Medical/Anatomy concept. Purple: Scientific community concept. Grey: Drug/Clinical trial concept. Each arrow describes the directionality of the dependency: From the parent scientific concept to the child scientific concept that depends on it. The L[N] represent the level of dependency depth of a scientific concept, i.e. the minimum number of dependency layer to comprehensively describe this scientific concept. A “P” on the bottom right corner describes a scientific concept which can be associated to publications, while a “E” describes annotated record by the Evidence and Conclusion Ontology.


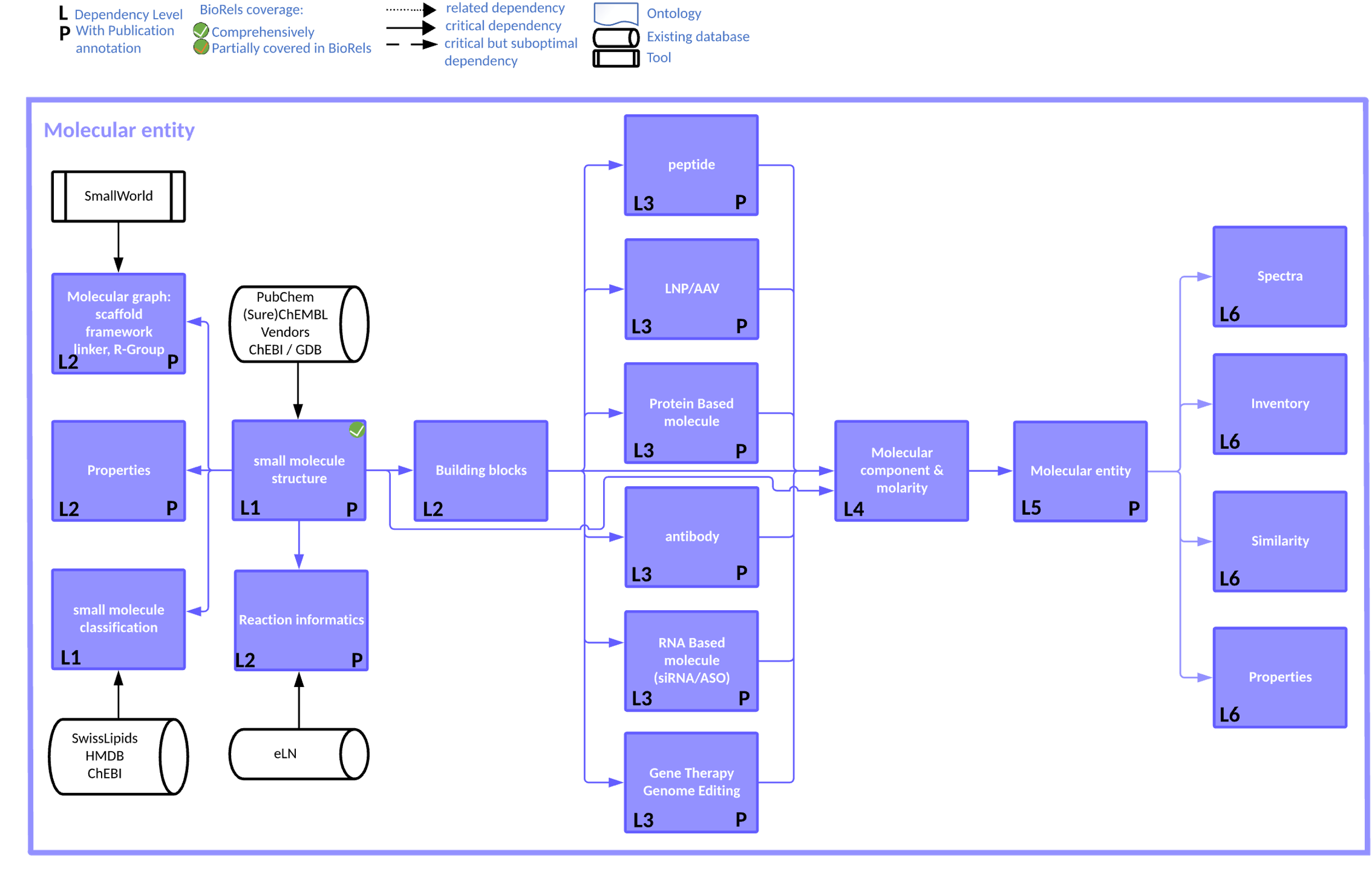


Supplementary Figure 4 Molecular entity ecosystem - Rounded Black: Potential public/private data source. Rectangular black box: tools. Melrose: Molecular entity scientific concept. Each arrow describes the directionality of the dependency: From the parent scientific concept to the child scientific concept that depends on it. The L[N] represent the level of dependency depth of a scientific concept, i.e. the minimum number of dependency layer to comprehensively describe this scientific concept. A “P” on the bottom right corner describes a scientific concept which can be associated to publications, while a “E” describes annotated record by the Evidence and Conclusion Ontology.


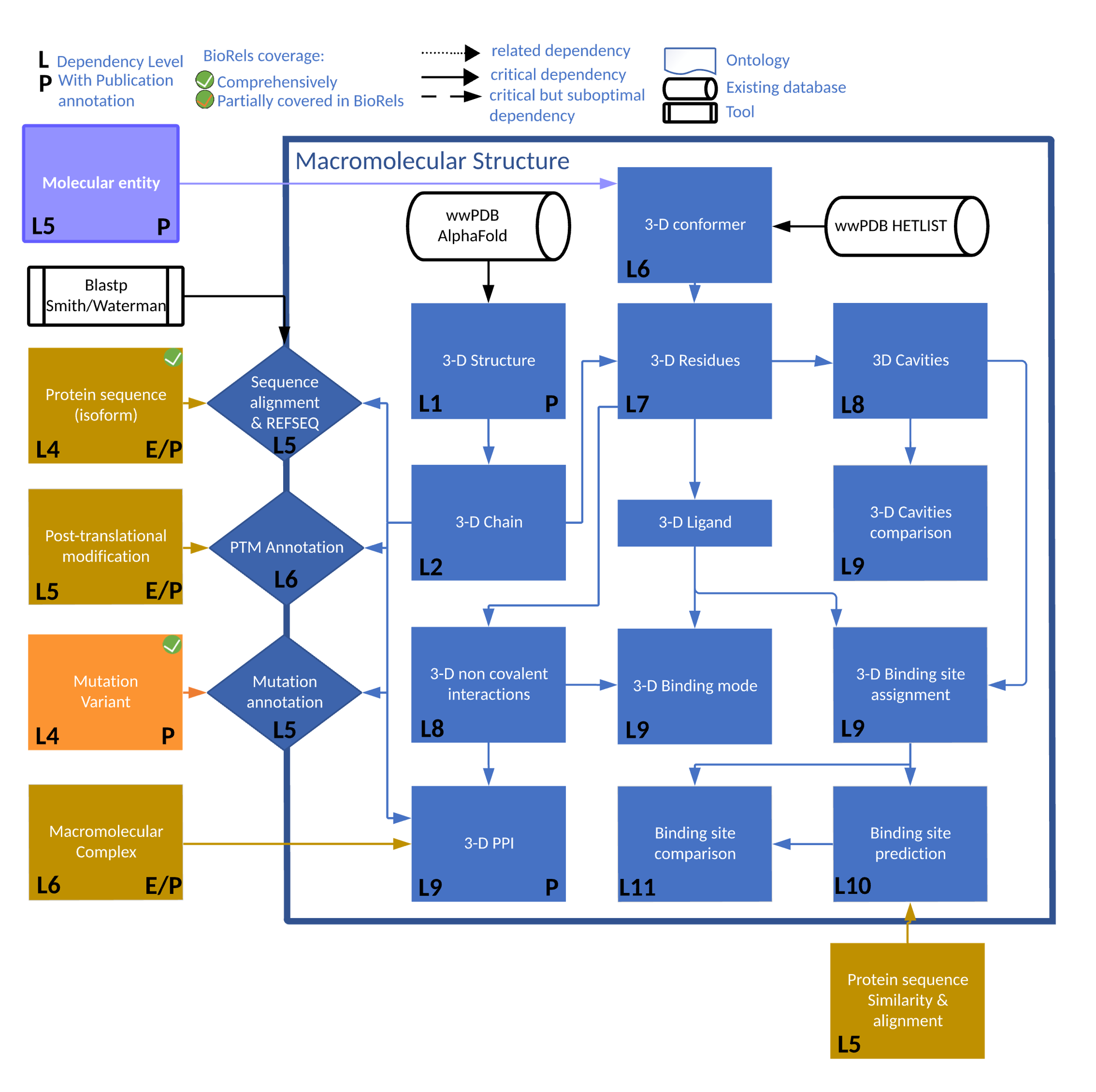


Supplementary Figure 5: Macromolecular Structure ecosystem - Rounded Black: Potential public/private data source. Rectangular black box: tools. Dark yellow: Proteomic scientific concept. Melrose: Molecular entity scientific concept. Orange: Genomic scientific concept. Blue: 3-D Structure concept. Each arrow describes the directionality of the dependency: From the parent scientific concept to the child scientific concept that depends on it. The L[N] represent the level of dependency depth of a scientific concept, i.e. the minimum number of dependency layer to comprehensively describe this scientific concept. A “P” on the bottom right corner describes a scientific concept which can be associated to publications, while a “E” describes annotated record by the Evidence and Conclusion Ontology.


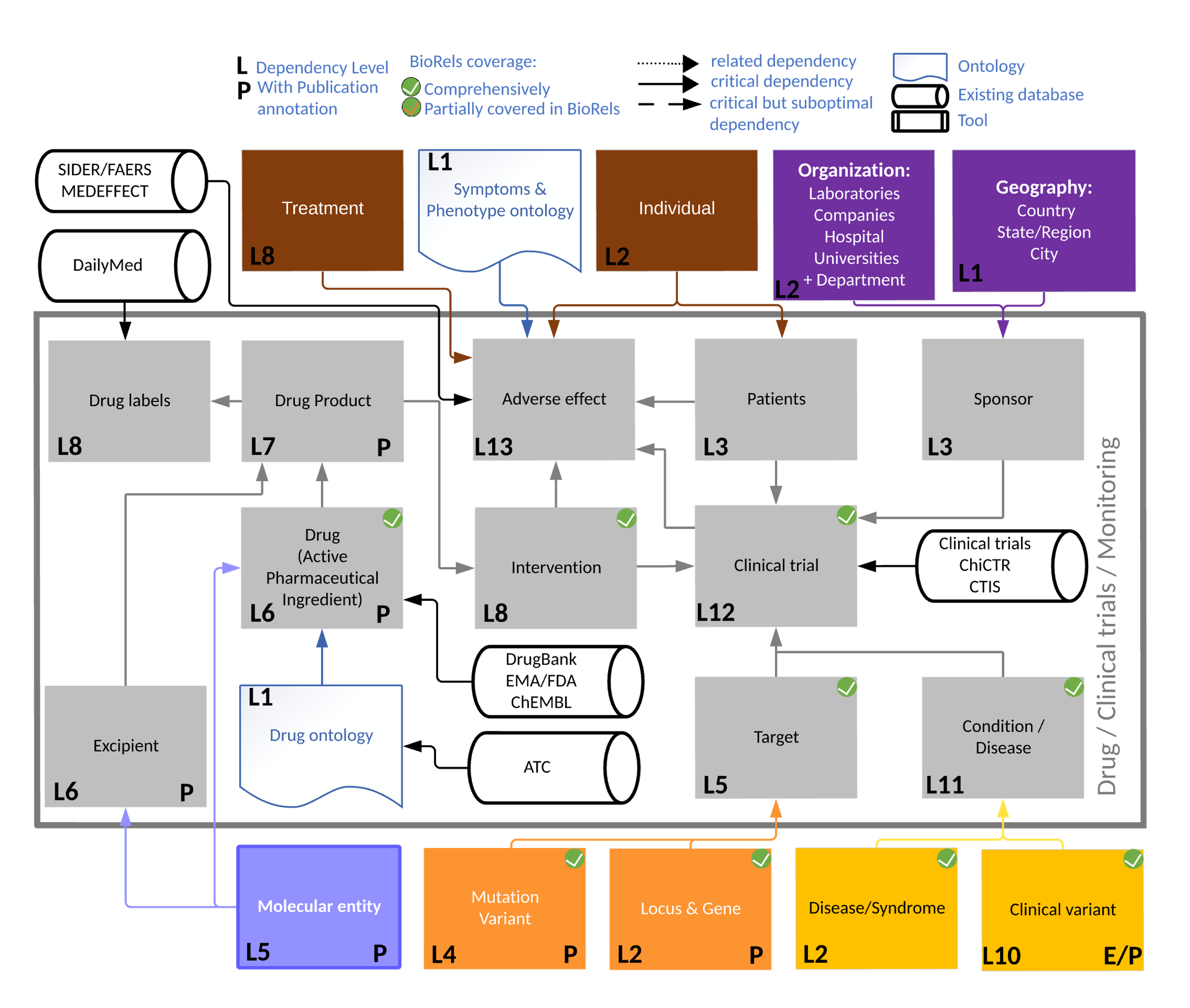


Supplementary Figure 6 Drug/Clinical trials/Monitoring ecosystem - Rounded Black: Potential public/private data source. Light blue shape: Ontology. Melrose: Molecular entity scientific concept. Orange: Genomic scientific concept. Yellow: Disease/Anatomy concept. Purple: Scientific community concept. Grey: Drug/Clinical trial concept. Dark yellow: Proteomic concept. Each arrow describes the directionality of the dependency: From the parent scientific concept to the child scientific concept that depends on it. The L[N] represent the level of dependency depth of a scientific concept, i.e. the minimum number of dependency layer to comprehensively describe this scientific concept. A “P” on the bottom right corner describes a scientific concept which can be associated to publications, while a “E” describes annotated record by the Evidence and Conclusion Ontology. Acronyms: ATC - Anatomical Therapeutic Chemical ; EMA – European Medicine Agency; FDA – Food and Drug Administration


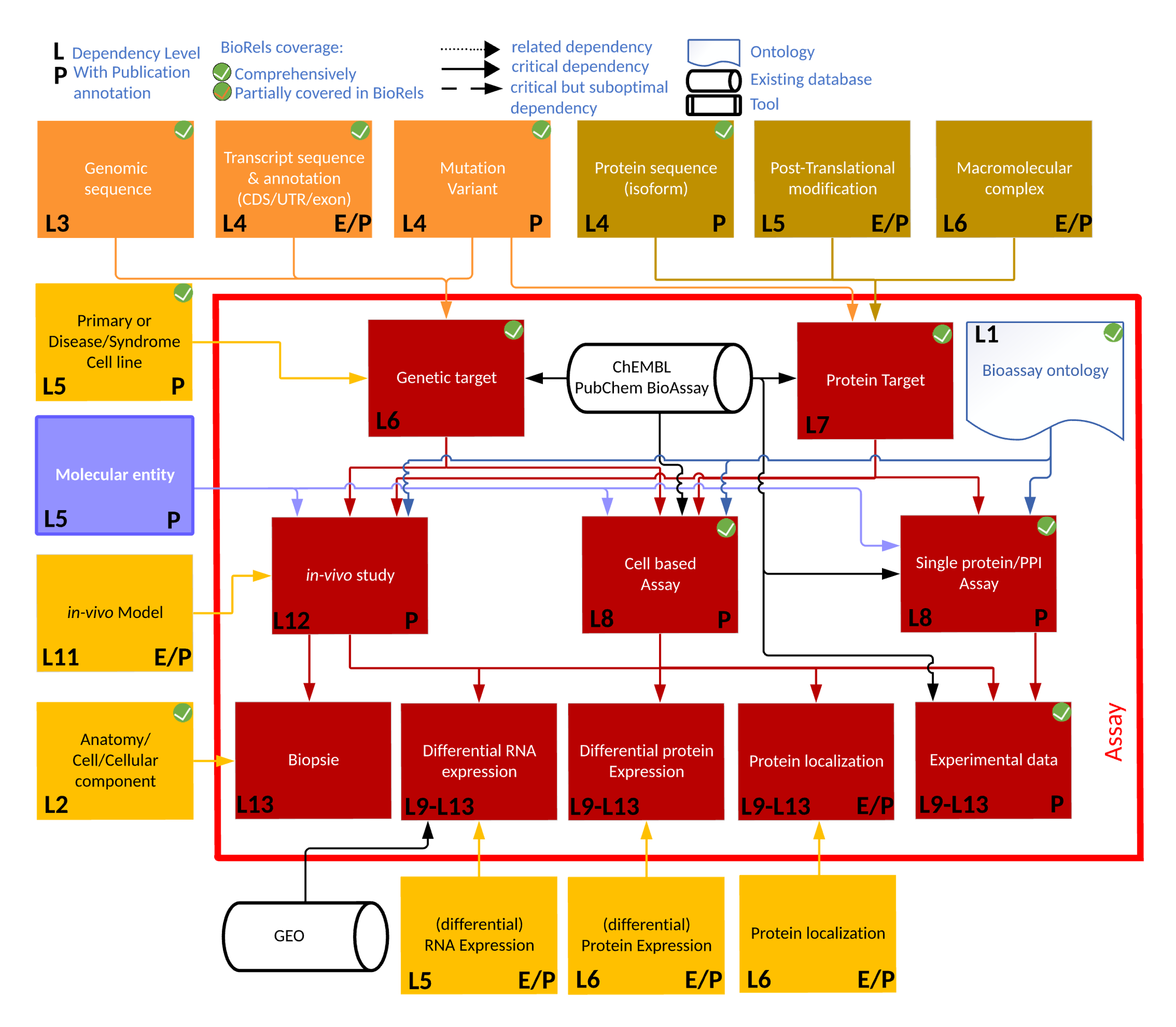


Supplementary Figure 7 Assay ecosystem: - Rounded Black: Potential public/private data source. Light blue shape: Ontology. Dark yellow: Proteomic scientific concept. Melrose: Molecular entity scientific concept. Orange: Genomic scientific concept. Yellow: Disease/Anatomy concept. Each arrow describes the directionality of the dependency: From the parent scientific concept to the child scientific concept that depends on it. The L[N] represent the level of dependency depth of a scientific concept, i.e. the minimum number of dependency layer to comprehensively describe this scientific concept. A “P” on the bottom right corner describes a scientific concept which can be associated to publications, while a “E” describes annotated record by the Evidence and Conclusion Ontology.


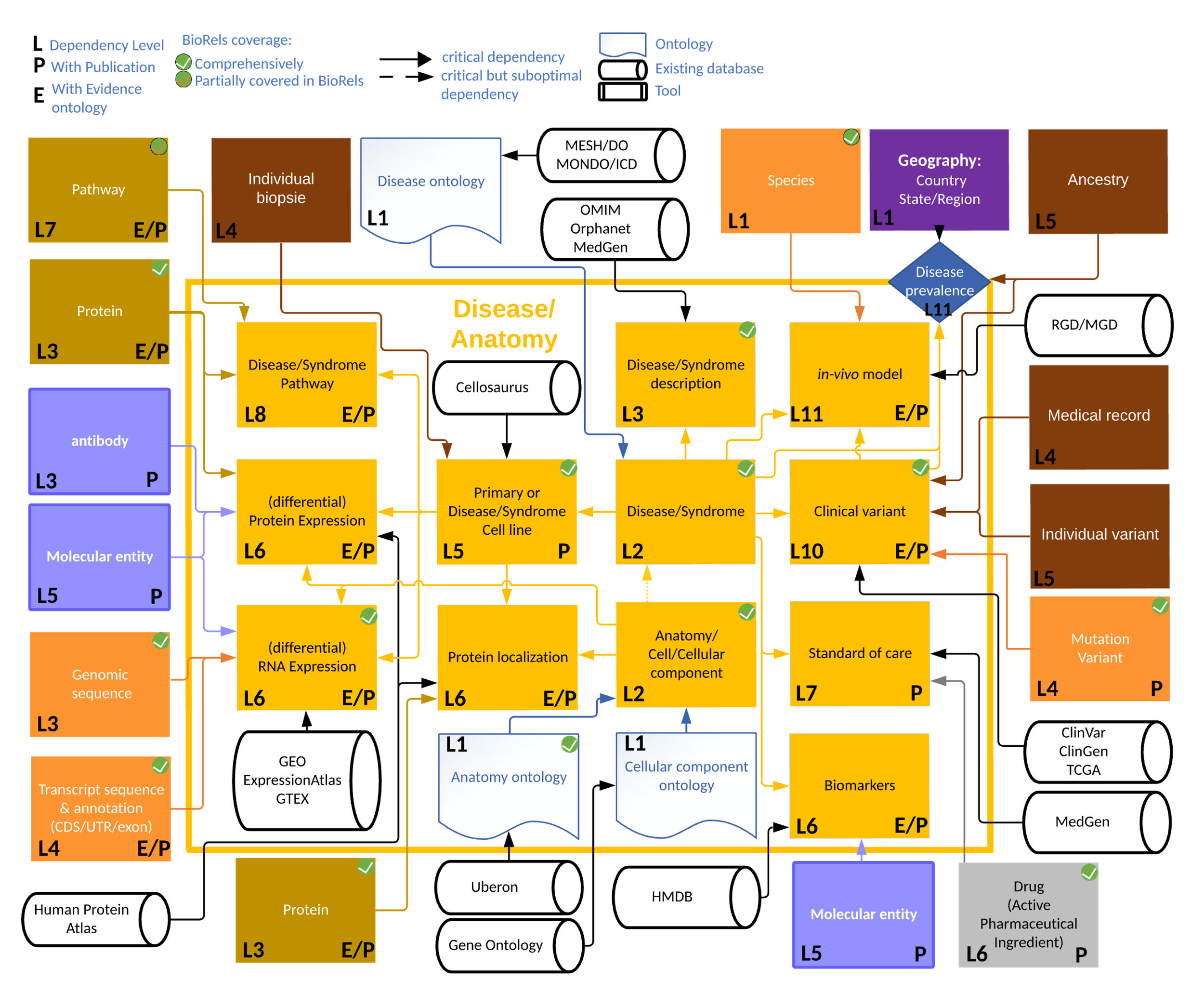


Supplementary Figure 8 Disease/Anatomy ecosystem: - Rounded Black: Potential public/private data source. Light blue shape: Ontology. Dark yellow: Proteomic scientific concept. Melrose: Molecular entity scientific concept. Orange: Genomic scientific concept. Brown: Medical concept. Yellow: Disease/Anatomy concept. Each arrow describes the directionality of the dependency: From the parent scientific concept to the child scientific concept that depends on it. The L[N] represent the level of dependency depth of a scientific concept, i.e. the minimum number of dependency layer to comprehensively describe this scientific concept. A “P” on the bottom right corner describes a scientific concept which can be associated to publications, while a “E” describes annotated record by the Evidence and Conclusion Ontology.


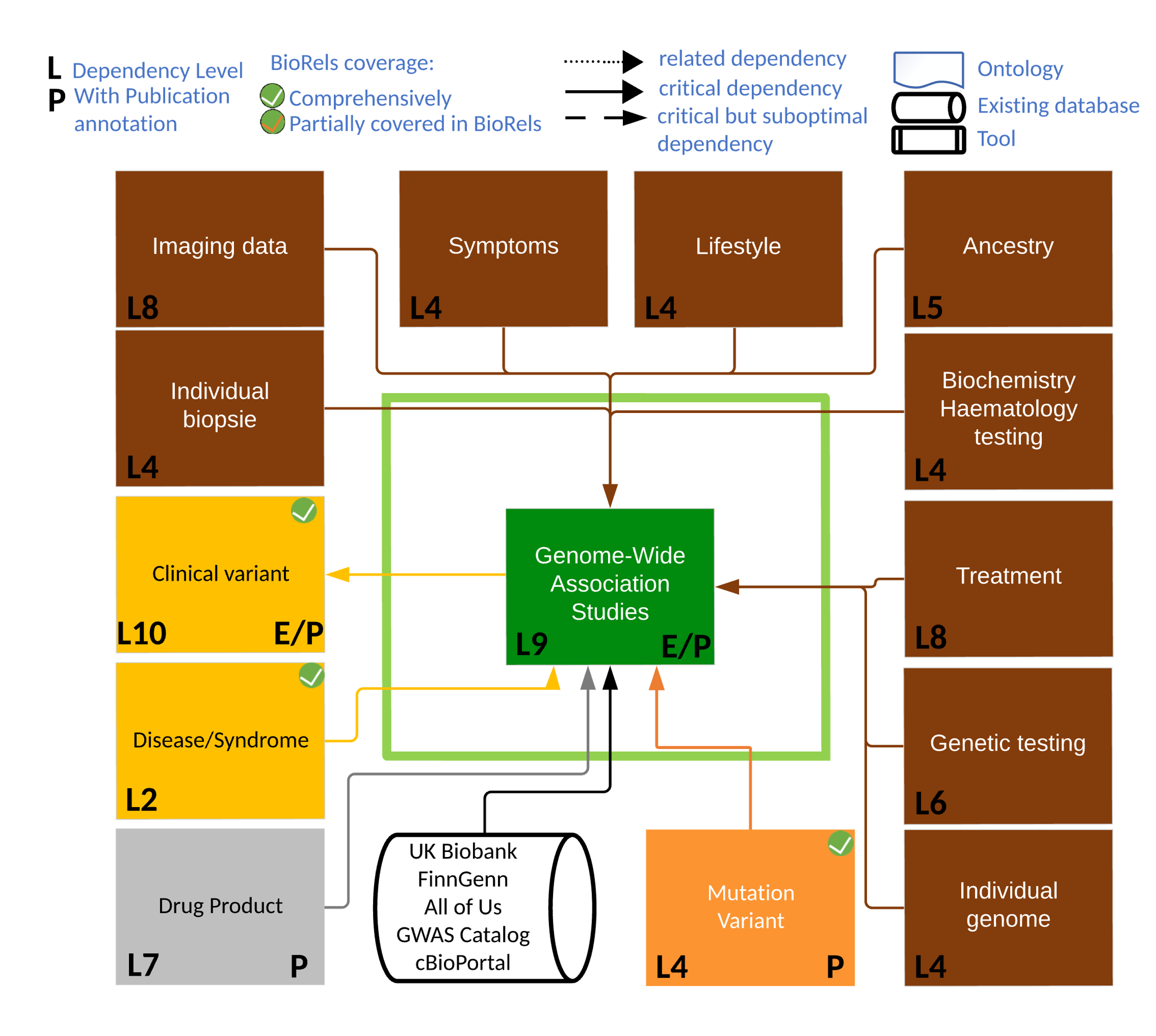


Supplementary Figure 9 Genome-Wide association study ecosystem: - Rounded Black: Potential public/private data source. Grey: Drug/Clinical trial concept. Orange: Genomic scientific concept. Yellow: Disease/Anatomy concept. Brown: Medical. Each arrow describes the directionality of the dependency: From the parent scientific concept to the child scientific concept that depends on it. The L[N] represent the level of dependency depth of a scientific concept, i.e. the minimum number of dependency layer to comprehensively describe this scientific concept. A “P” on the bottom right corner describes a scientific concept which can be associated to publications, while a “E” describes annotated record by the Evidence and Conclusion Ontology.


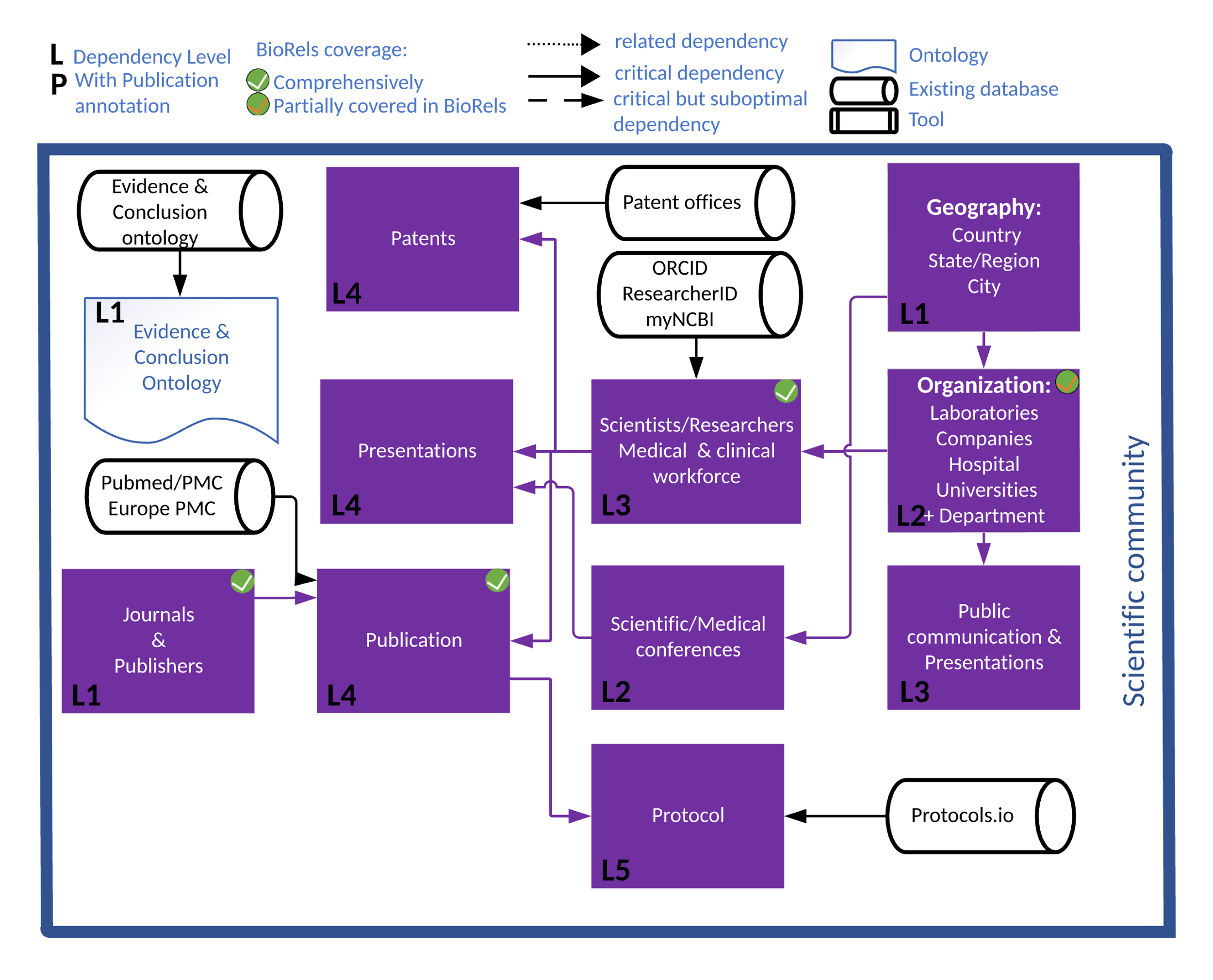


Supplementary Figure 10 Scientific community ecosystem: - Rounded Black: Potential public/private data source. Light blue shape: Ontology. Purple: Scientific community. Yellow: Disease/Anatomy concept. Each arrow describes the directionality of the dependency: From the parent scientific concept to the child scientific concept that depends on it. The L[N] represent the level of dependency depth of a scientific concept, i.e. the minimum number of dependency layer to comprehensively describe this scientific concept. A “P” on the bottom right corner describes a scientific concept which can be associated to publications, while a “E” describes annotated record by the Evidence and Conclusion Ontology.

## Supplementary text 1 – Complete description of Biorels data preparation processes.

**Taxonomy:**

First, all organisms, with their NCBI Taxonomy Identifier, name and rank, are taken from NCBI ftp server^1,2^ by selecting only entries with name class is “scientific name”. In addition to their name, their taxonomy lineage is generated by converting the organism’s relationships into a nested set representation. Taxonomic data is stored in the TAXON table and their relationships in TAXON_TREE.

**Gene:**

Gene information for all known genes is downloaded from NCBI Gene FTP server^3^. First, all obsoletes gene records, as provided by gene_history, are removed from the database. Gene records for which their NCBI gene Identifier has been changed will be updated. In addition, the user has the possibility to limit the processing to a list of pre-defined organisms, characterized by their NCBI taxonomic ID. For each processed record, symbol and name from nomenclature authority will be preferred to NCBI values. Symbol, full name, gene type and status are stored in the gene entry table (GN_ENTRY). From there, the association between a gene and an organism is performed via three tables: Chromosome (CHROMOSOME), cytogenetic location (CHR_MAP) and a mapping table between the cytogenetic location and gene record (CHR_GN_MAP).

Next, all chromosomes reported in the chromosome field are considered and stored in the chromosome table (CHROMOSOME). Values such as Un, undetermined, Unknown or – are recorded as “Un”.

Then, cytogenetic location defined in map_location field is processed. Text such as “+/-“ is removed and split by fields like “and”, “|”, “;” or space. All centimorgan values or text such as “–“, A, B, C, D, E, F,G,H, alternate, h, tbd or L are considered as Unknown location. In this event, an Unknown (Un) record will be created for each chromosome reported in the chromosome field for this record. Otherwise, each cytogenetic location is broken down into the position (alias to chromosome), region, band and sub-band and save in the CHR_MAP table. When multiple locations are provided for a given gene, all locations are considered. Once done, the connection between those cytogenetic locations and the gene record is recorded in CHR_GN_MAP table.

Gene symbol, aliases and alternative names from both the nomenclature authority and NCBI gene are also extracted and uniquely stored in a table of synonym (GN_SYN) to enable search capabilities. GN_SYN table distinguish symbols from names by setting the *syn_type* column value to S or N respectively. The mapping between these aliases and gene entries is made possible thanks to GN_SYN_MAP table.

In addition to gene information, the list of gene orthologous pairs is also retrieved from NCBI Gene FTP server^3^ and stored in GN_REL table. It is however augmented with the list provided by HCOP^4,5^ from HGNC.

**Reactome Pathway:**

Pathway information is also incorporated by taking advantage of the Reactome database^6^. Every pathway name and identifier corresponding to a specific taxon are taken from ReactomePathways.txt and saved in PW_ENTRY table. A manual correction is applied to Canis familiaris taxon that is stored as Canis lupus familiaris in the database. Since Reactome organized their pathway hierarchically, a nested set representation identical to the one used for taxonomy is generated using ReactiomPathwaysRelation.txt file. Pathways are then associated to their corresponding gene entries by pushing NCBI2Reactome.txt data into PW_GN_ENTRY table.

**Gene Ontology:**

Protein functions, biological processes and cellular location for each gene is incorporated in the database by integrating information generated by the Gene Ontology consortium^7,8^. GO term, name, namespace, definition, comment and status are stored for each record in the GO_ENTRY table Relationships between those GO entries, such as is_a or inverse_of for example, are pushed in the GO_REL table. Publications are extracted using regular expressions from the annot_def and synonym fields and saved in GO_PMID_MAP table. All synonyms as defined in the synonym field are stored in GO_SYN table. For external identifiers, all external sources are first mapped to the SOURCE table and created if missing. External identifiers are then connected to their source and respective GO entry in the GO_DBREF table.

**Sequence Ontology:**

Sequence Ontology is downloaded from <https://raw.githubusercontent.com/The-Sequence-Ontology/SO-Ontologies/master/Ontology_Files/so.obo> from which the id, name and def fields are extracted, population SO_ID, SO_NAME and SO_DESCRIPTION fields in SO_ENTRY table.

**UBERON Ontology:**

Similarly to the sequence Ontology, UBERON ontology is downloaded in obo format. Class set as a gene_symbol_report, deprecated or without any name are discarded. Name, label and identifier are extracted for each class and stored in the ANATOMY_ENTRY table. All external identifiers defined in oboInOwl:hasDbXref field are retrieved and the source of those identifiers are tested against and saved in the SOURCE table. External identifiers are then saved in the table ANATOMY_EXTDB. Exact, Broad, Related, Narrow synonyms are recorded in the ANATOMY_SYN table. At last, the hierarchy between the different ontology entries are represented thanks to a nested set representation in ANATOMY_HIERARCHY.

**BioAssay Ontology:**

Bioassay Ontology is downloaded in obo format. We only consider records with an identifier starting with the BAO_ prefix and not deprecated. Name and description for each record is retrieved by taking the content in rdfs:label and obo:IAO_0000115 xml tags, respectively. Parent to child relationship is extracted from rdfs:subClassOf tag, unless the parent is owl#Thing. Name, description and identifier are saved in BIOASSAY_ONTO_ENTRY. The hierarchy between the different ontology entries are represented using a nested set representation in BIOASSAY_ONTO_HIERARCHY.

**Cellausorus:**

Cell lines are also incorporated in the database by taking advantage of the Cellausorus database. The cell name, accession, type, donor’s sex and age of each cell line is saved in CELL_ENTRY table. Each cell line record provides additional information, such as the patent or publication they have been initially reported, the organism, tissue and disease state they have been extracted from. Thus, lookup queries are performed against PMID_ENTRY (publication), PATENT_ENTRY (patent), ANATOMY_ENTRY (tissue), DISEASE_ENTRY (disease) and the corresponding connections are stored in CELL_PMID_MAP, CELL_PATENT_MAP, CELL_TISSUE, CELL_DISEASE tables, respectively. If a patent is not present in the PATENT_ENTRY table, the record will be created prior to adding the corresponding connection in CELL_PATENT_MAP.

**Evidence and Conclusion Ontology (ECO)**

The evidence and conclusion ontology, generated by EBI, is downloaded in obo format at http://purl.obolibrary.org/obo/eco.owl. Label (rdfs:label), description (obo:IAO_0000115) and ECO identifiers are retrieved from the input file and saved in ECO_ENTRY table. In contrast with taxonomy in which there is only one lineage per species, an ECO record can follow multiple hierarchy of ontology. To consider them all, a nested set representation is generated and stored separately from ECO_ENTRY in ECO_HIERARCHY table.

**Experimental Factor Ontology**

The Experimental Factor Ontology is retrieved from <http://www.ebi.ac.uk/efo/efo.owl>. The ID – contained in the owl:Class line, the label defined in rdfs:label and the definition from obo:IAO_0000115 are retrieved for each record and stored in EFO_ENTRY tables as EFO_TAG_ID, EFO_LABEL and EFO_DEFINITION columns. External identifiers retrieved from oboInOwl:hasDbXref lines are stored in EFO_EXTDB table. Because an EFO record can be associated to one or multiple parents as defined by rdfs:subClassOf lines, a nested set representation is generated and stored in EFO_HIERARCHY.

**MONDO Ontology – Disease**

The MONDO disease ontology is retrieved from <http://purl.obolibrary.org/obo/mondo.owl>. Similarly to EFO ontology, the ID – contained in the owl:Class line, the label defined in rdfs:label and the definition from obo:IAO_0000115 are retrieved for each record and stored in DISEASE_ENTRY tables as DISEASE_TAG, DISEASE_NAME and DISEASE_DEFINITION columns. External identifiers retrieved from oboInOwl:hasDbXref lines are stored in DISEASE_EXTDB table. Synonyms are retrieved from oboInOwl:hasExactSynonym, oboInOwl:hasRelatedSynonym, oboInOwl:hasNarrowSynonym, oboInOwl:hasBroadSynonym and stored in DISEASE_SYN table.

**Publication**

First, the list of scientific journals is downloaded from PubMed ftp server^9^, and processed to insert the MedAbbr, ISSN (Print), ISSN (Online), Iso Abbreviation, NLM Identifier and journal title for each journal in PMID_JOURNAL. Each PubMed xml file with a creation date later than the last processed date is downloaded from PubMed baseline and updatefiles ftp directories. Then, each publication record is processed as follow. First, publication title, abstract, date, Doi, PMID, volume, pages and journal are recorded in PMID_ENTRY table. If the journal is not present in PMID_JOURNAL, it will be first added prior to adding the publication. Then, all authors institutions are retrieved and inserted in PMID_INSTIT table if they do not exist. Following this, each author is checked for duplicate in the PMID_AUTHOR by looking at the first and last name, initials, institution and the ORCID ID when it is provided. An author and its information are inserted only if not found the table. PMID_AUTHOR_MAP records the connection between a publication and its authors, with their added position in the list of authors.

**RefSeq**

The list of organisms to be considered are defined by the user in the CONFIG_USER file. For each species, the process is defined as followed. First, files with the suffix _assembly_report, _genomic.fna, _rna.fna and _genomic.gff are downloaded from RefSeq server. If provided, the RefSeq transcripts alignment files are also downloaded for both known and model RefSeq entries. samtools view is run against each alignment file to extract their headers.

**Ensembl**

Similar to RefSeq, only organisms defined by the user in the CONFIG_USER file will be considered. We first check for a new Ensembl release by downloading the current_README from Ensembl ftp website. If a new version is available, all files with the following suffix are downloaded: _assembly.txt, _dna_sm.toplevel.fa.gz, _all.fa.gz, _ncrna.fa.gz and _gff3.gz. For Homo Sapiens, we chose to download the patch_hapl_scaff gff file.

**Genomic sequence**

Once all Ensembl and Refseq files are downloaded for the selected organisms, a selection is performed based on user preference. A user can select to use either the source that uses the most current assembly or both sources. To assess the assembly, the RefSeq assembly_report as well as Ensembl assembly file are processed to extract the GenBank assembly accession value. The complete assembly information from both RefSeq and Ensembl are downloaded in xml format using NCBI assembly ftp server. We then extract the AsmReleaseDate_GenBank date and compare the values for both data sources. In the event where a user only wants to consider the latest assembly, if the GenBank release date is the same for both sources, then both sources will be considered. Otherwise, only the source with the latest GenBank release date will be incorporated. From there, the assembly accession number (GCF number), its version, assembly name, creation data, last update date as well as their connection to the TAXON table are stored in the GENOME_ASSEMBLY table.

Next, chromosome sequence information is loaded into CHR_SEQ table. To do so, the RefSeq assembly file is processed to extract for each chromosome sequence the RefSeq name and version, the GenBank name and version, the sequence role, assembly unit as well as its connection to the genome assembly and chromosome record. Ensembl assembly file is then processed if selected. In the event where both sources are considered, only chromosome sequences not already covered by RefSeq will be added. If only Ensembl is considered, all chromosome sequences will be processed and stored in CHR_SEQ. For localized scaffold and patches, their starting and ending position within the chromosome are added using Ensembl gff file. The DNA sequence of each chromosome, including scaffold and patches is then stored in CHR_SEQ_POS. To do so, each nucleotide, its position as well as its corresponding chromosome sequence record as provided in the _genomic.fna is loaded. The position of each nucleotide is based on the sequence and not the eventual starting position if the sequence is a localized patch or scaffold. To ensure validity of a given chromosome sequence, its length and an md5 hash are computed and compared to the sequence from the input file.

A difference is made between a gene record as defined by NCBI Gene and saved in GN_ENTRY and its location in the corresponding chromosome stored in GENE_SEQ. Therefore, for each chromosome, all gene sequences as defined by gene (RefSeq & Ensembl), pseudogene (RefSeq & Ensembl) or ncRNA_gene (Ensembl), including those located on patches or scaffolds are extracted with their metadata (strand, starting position, biotype, feature) and stored in GENE_SEQ. While the connection between GN_ENTRY (NCBI Gene) and Gene sequences is already present in RefSeq GFF files, an additional step using gene2ensembl mapping file from NCBI is performed for Ensembl Gene Sequences.

Furthermore, transcript information is analyzed by processing gff files. First, only lines within a gene, pseudogene or ncRNA_gene sequence range are considered. Then, all transcripts with the following types are selected for further process: C_region, D_segment, IG_C_gene, IG_C_pseudogene, IG_D_gene, IG_D_pseudogene, IG_J_gene, IG_J_pseudogene, IG_LV_gene, IG_pseudogene, IG_V_gene, IG_V_pseudogene, J_segment, lncRNA, miRNA, misc_RNA, mRNA, Mt_rRNA, Mt_tRNA, ncRNA, nonsense_mediated_decay, non_stop_decay, polymorphic_pseudogene, precursor_RNA, processed_pseudogene, processed_transcript, protein_coding, pseudogene, retained_intron, ribozyme, rRNA, rRNA_pseudogene, scaRNA, scRNA, snoRNA, snRNA, sRNA, TEC, transcribed_processed_pseudogene, transcribed_unitary_pseudogene, V_segment, Y_RNA, transcribed_unprocessed_pseudogen, translated_processed_pseudogene, translated_unprocessed_pseudogene, TR_C_gene, TR_D_gene, TR_J_gene, TR_J_pseudogene, tRNA, TR_V_gene, TR_V_pseudogene, unitary_pseudogene, unprocessed_pseudogene, vault_RNA. The support level is defined by Ensembl by a numerical value ranging from 1 (most confident) to 5 (least confident). For RefSeq transcripts, support level value is set to 1 and 5 when the transcript name starts with N and X respectively. If the sequence is a partial sequence as noted in gff attributes, the partial_sequence field is set to T. The corresponding transcript name, version, start and end positions, gene sequence, biotype and support level are extracted from the gff file and saved into the TRANSCRIPT table. For micro-RNA transcripts, the support level is based off the parent mRNA while for tRNA the support level is left blank. Once a transcript has been annotated, its sequence is then built from the DNA sequence based off the exon annotations, except for tRNA, C_region, D, J and V segments, which have no sequence provided by RefSeq/Ensembl. To do so, we retrieve for a RefSeq transcript its alignment against DNA sequence using PySAM module and provided BAM files. For Ensembl transcript, a simple 1-to-1 mapping with the DNA is performed. Using the gff information related to that transcript, each nucleotide is then annotated by its position within the chromosome, the exon number and the coding type: 3’ UTR, CDS, 5’ UTR. In addition, the alignment is used to annotate insertion, deletion and poly-A. Only Adenosine nucleotides at the end of the transcript sequence for which no chromosomal position nor exon have be assigned to are set to poly-A. When the chromosomal position and exon number are not found for a given transcript nucleotide, the coding region is inferred from neighboring nucleotide’s annotation by using the following rules: 1/ If the annotations before and after are the same, report that annotation + ‘-INFERRED’ appended to the end (i.e. ‘CDS-INFERRED’, ‘5’-UTR-INFERRED’, etc.). 2/ If the annotation before is 5’UTR and the annotation after is CDS, report 5’UTR-INFERRED because the start codon is in the CDS so anything before that is still 5’. 3/ If the annotation before is CDS and the annotation after is 3’UTR, report CDS-INFERRED because the stop codon is in the 3’UTR so anything before that is still CDS. 4/ If the annotation before is 3’UTR and the annotation after is poly-A, report 3’UTR-INFERRED because if it wasn’t already flagged as a poly-A tail then it is an extension of the 3’UTR. 5/ If the annotation before is non-coded and the annotation after is poly-A, report non-coded-INFERRED because if it wasn’t already flagged as a poly-A tail then it is an extension of the transcript. 6/ If the annotation before doesn’t exist (start of sequence) or the annotation after doesn’t exist (end of sequence) report the existing, adjacent annotation + ‘-INFERRED’. 7/ Any other scenarios will get reported as unknown which may cover cases of partial sequences. If the alignment between the transcript and the DNA has more than 5% of mismatches and is not considered to be a partial sequence, the sequence is subsequently ignored. To ensure integrity of a given sequence, a md5 hash is created based on the individual nucleotide, their position, exon id and annotation to ensure a complete identification of the sequence and its annotation. If the md5 hash is different from the one in the database, the transcript sequence is deleted, including the connected data, and replaced by the new sequence.

**Uniprot:**

Prior to any Uniprot entry processing, the reference for external databases is first downloaded for Uniprot ftp server (dbxref.txt). Each external database record is processed and saved in PROT_EXTDB table. The list of proteomes and the inclusion of Swiss-Prot and TrEMBL is defined by the user in the CONFIG_USER file. Due to the size of TrEMBL, only records associated to a 3-D structure or an ChEMBL assay are considered. The association between a used-provided NCBI taxonomy Identifier and a UniProt proteome is made by using the Uniprot proteome README file from the ftp server. This README associate NCBI Taxonomy ID, super-reign and Proteome ID, which are subsequently used to download the UniProt records (file with suffix .dat.gz), protein sequences (file with suffix .fasta.gz) as well as additional data (file with suffix _additional.dat.gz and _additional.fasta.fz). All sequence files are concatenated into an ALL_SEQ.txt file. A blast database is then built using makeblastdb and ALL_SEQ.txt file as input. SwissProt records are provided in UniProt ftp server by downloading uniprot_sprot.dat.gz (SwissProt records), uniprot_sprot.fasta.gz (fasta sequences) and uniprot_sprot_varsplic.fasta.gz (fasta isoform sequences). A blast database using makeblastdb is created using a concatenated fasta file of both primary and isoform sequences. In a similar process, TrEMBL records (uniprot_trembl.dat.gz) and protein sequences (uniprot_trembl.fasta.gz) are downloaded from the ftp server and a blast database is created from the protein sequence fasta file. Protein information is then stored in the database via various tables. Each entry is uniquely defined by its Uniprot Identifier in the PROT_ENTRY table. Taxonomy is assigned using the OX (Organism Taxonomy cross-reference) line and linked to the TAXON table based on NCBI Tax ID. All Accession identifiers are listed from the AC lines and stored in PROT_AC table. Only the first accession in the first AC line will be considered as primary. In addition, the connection between UniProt entry and Gene entry, stored in GN_PROT_MAP is made by using the Gene ID value (DR GeneID). Gene selection is further confirmed by using when available the Ensembl gene name (DR Ensembl), and the Uniprot official Gene Name (GN section) in combination with the TaxID provided in the Uniprot Entry. However, no connection is made if the gene name exist in multiple gene entries within the same organism either as a primary symbol or an alias. In addition, UniProt provide connection between its record and Gene Ontology entries, which are extracted to populate the PROT_GO_MAP table. Each protein name and eventually Enzymatic Commission number provided in the DE lines are processed individually are stored in the PROT_NAME table. The association between the protein names and UniProt identifiers is then made through PROT_PNAME_MAP table that describes whether the protein name is the recommended or the alternative name (NAME_TYPE), whether it is the full name or a synonym (NAME_SUBTYPE), whether it represent the inclusion of a functional domain (NAME_CLASS).

For a given UniProt record, all sequences including their isoforms are recorded in the database into PROT_SEQ and PROT_SEQ_POS tables. First, the alternative products field of the comment section (CC lines) is processed to retrieve the Isoform Identifier (IsoID) and the sequence name (Sequence). A sequence name set to Displayed is defined as the canonical sequence (is_primary=T) while other sequences will be considered as alternative isoform. The description is derived from the fasta header of the isoform, while a sequence note is built from the concatenation of all VAR_SEQ features associated to it. Each amino acid of each sequence is stored individually and separately with their position in the UN_SEQ_POS table. Among the many sequence features provided by Uniprot, CHAIN, DOMAIN, REPEAT and REGIONS derived from the canonical sequence are retained and stored in UN_DOM. Each amino acid within the feature sequence range is individually stored in UN_DOM_POS table. However, instead of recording the position like in the UN_SEQ_POS, we here point each amino acid of a domain sequence to itself in the UN_SEQ_POS table.

All protein features listed in FT lines are then processed. By default, all features are associated to the canonical sequence, unless explicitly specified. The location (start/end positions), the value (note section) and type of each feature are saved in PROT_FEAT table. Each association between each amino acid and a feature is saved in PROT_FEAT_SEQ table. When provided, the PubMed identifier as well as the ECO identifier are associated to a protein feature in PROT_FEAT_PMID.

Chain, region, domain and repeat are extracted from protein features if their location is clearly defined (no question mark in the location field) and saved with the feature name and type in PROT_DOM table. In the event the starting or ending position is defined as greater or lower than a given position, only that position will be considered. The corresponding sequence is extracted from the canonical sequence and saved into PROT_DOM_SEQ.

The Database cross-reference lines (DR Lines) are then processed for a given record. For each cross-reference, the external database is mapped to itself in the PROT_EXTDB table. The external database identifier is extracted and if applicable, is associated to a given protein isoform from the PROT_SEQ table. All records are then saved in PROT_EXTDB_MAP table.

**InterPro**

BioRels also include a more precise domain and protein family definition generated by InterPro^10^. Data is first downloaded from InterPro ftp server: interpro.xml.gz, match_complete.xml and tree.txt. The short name, protein count, entry type, name and abstract of each InterPro entry is then extracted from interpro.xml and saved in IP_ENTRY table. Publications, GO entries, and external database identifiers are extracted and stored in IP_PMID_MAP, IP_GO_MAP and IP_EXT_DB tables, respectively. Then, tree.txt is processed to create the InterPro hierarchy that is converted into a nested set representation in IP_ENTRY. In addition, each InterPro signature, which are prediction from InterPro member databases, is extracted from interpro.xml and its source member, identifier and name are saved in IP_SIGNATURE table. Using match_complete.xml file, each protein sequence from UniProt is associated to the corresponding InterPro record thanks to IP_PROT_SEQ_MAP table**.**

**Protein sequence alignment**

Protein sequence similarity is initially done by exporting from the database all protein sequences, including isoforms in a fasta format. In order to keep the database size manageable, only Human sequences are compared against all sequences using first blastp^11^ with default parameters. To avoid keeping in the database irrelevant matches, pairs found by blastp are discarded if their percent identity is below 30%. In addition, a pair is removed if the ratio matched sequence length vs reference sequence length is below 70% when the reference sequence length is lower than 300 amino acids or 80% if higher than 300 amino acids. Each remaining pair of matched sequences are aligned using Smith-Waterman sequence alignment^12^ with BLOSSUM62^13^. The statistics of the alignment, including percentage of identity and similarity of the overall sequences and the aligned portion is stored in UN_SEQ_AL table. Each pair of amino acids defined by the alignment is stored in the database in UN_SEQ_AL_SEQ table.

Similarly to protein sequences, all feature sequences are extracted for alignment, with the additional requirement that the sequences at longer than 30 amino acids. Only Human feature sequences are compared to all other feature sequences. In addition, we also differentiate CHAIN feature from DOMAIN, REPEAT and REGIONS. In opposition to CHAIN feature where standard blastp^11^ parameters have been applied, DOMAIN, REPEAT and REGION have been aligned using the following constraints: -word_size=3 -max_hsps 1 -evalue 100. The protocol is then identical to the protein sequence alignment, with the exception that the storage in the database is respectfully done in UN_DOM_AL for the alignment statistics and UN_DOM_POS for the amino-acid pairs.

**mRNA translation to Protein**

While protein-coding transcripts are connected to their corresponding UniProt records via external identifiers, the translation mRNA to protein is also pre-computed. For each gene, all protein isoforms as well as transcripts having CDS tags in the TRANSCRIPT_POS table, i.e. the transcript sequence, are downloaded. All CDS regions of those transcripts are then translated using transeq and an in-house smith waterman sequence alignment is performed against protein isoforms. Only alignments with more than 99% sequence identity are retained, unless the association has been reported in UniProt, and their statistics (% identity, % similarity) are stored in TR_UNSEQ_AL table. The nucleotide to protein mapping is also saved in the TR_UNSEQ_AL_POS table, with the additional information of the nucleotide position in the codon.[ <https://www.ebi.ac.uk/seqdb/confluence/display/JDSAT/EMBOSS+Transeq+FAQ#EMBOSSTranseqFAQ-HowdoIreferenceuseofEMBOSSTranseqattheEBI>?]

**RNA Expression**

RNA Expression data is manually downloaded from the GTEX portal v8 in <https://gtexportal.org/home/datasets>. First, Samples are associated to their corresponding UBERON or EFO tissues using a manual annotation map and saved in RNA_SAMPLE. Then, each transcript is matched against TRANSCRIPT table and the association transcript/sample/Expression value in TPM is saved in RNA_TRANSCRIPT. Then statistics (minimal TPM value, first quartile, median, third quartile, maximal value, number of samples) for each pair of transcript and tissue is computed and stored in RNA_TRANSCRIPT_STAT. To understand the selectivity of a given transcript for a given tissue, a Receiver Operating Characteristic curve (ROC curve) is generated and its Area under curve (AUC) computed by rank ordering the TPM value decreasingly and considering the looked-up tissue as positive and the other tissues as negative. Gene TPM values are stored in RNA_GENE and a similar statistical process is performed and saved in RNA_GENE_STAT.

**dbSNP**

First, studies information is downloaded for dbSNP^14^ and ALFA^15^ and each individual study is saved in VARIANT_FREQUENCY_STUDY table. The json variant file is then downloaded for each chromosome from dbSNP ftp server^16^ and their integrity verified using the provided checksums. Several steps are then performed for each variant record. First, each RNA annotation withing the primary snapshot data are extracted along with its DNA position, the deleted and inserted sequences and the transcript name. In parallel, all frequency information, including the study name and version, the allele and total count for each inserted sequence within the allele annotations of the primary snapshot data are obtained. Then, each variant type within the primary snapshot data is processed if the sequence type is refseq_chromosome and the placement annotation is using GrCh38. For each allele within a variant type, its position, deleted and inserted sequences as well as the sequence identifier are extracted, and its corresponding frequencies previously analyzed are added. At last, all genes within the assembly annotation section are processed and their associated transcripts analyzed. To do so, we first look if a protein product identifier is specified. If so, the protein name, sequence ontology accession identifier and position are extracted as well as the impact: whether it is a frameshift or a variant. In the latter case, the inserted and deleted amino acid is also included. Once all protein information has been considered, the sequence ontology accession describing the impact of that variant on the transcript is then analyzed. Thus, we distinguish between coding and non-coding change. Since the codon is provided in the case of a coding change, we only keep the location of the variant. For a non-coding change, if it is located within the UTR regions, we record the sequence identifier, position, deleted and inserted sequences that we associate to its protein impact previously described. Once all the information for a given record has been processed, each variant record is saved in VARIANT_ENTRY, its DNA location on chromosome or scaffold/patches and its reference allele are saved in VARIANT_POSITION. Each alternative allele is stored in VARIANT_CHANGE, while the reported frequencies are saved in VARIANT_FREQUENCY. At last, the impact on the transcript and eventually the protein is saved in VARIANT_TRANSCRIPT_MAP and VARIANT_PROTEIN_MAP respectively. ALFA frequencies are then individually processed, by associating them to their corresponding variant and added to VARIANT_FREQUENCY table.

**Open Targets**

Molecule, evidence, indication, mechanism of action and disease to phenotype json files from OpenTargets^17^ are downloaded from their ftp server^18^. Currently, only evidence from EuropePMC and ChEMBL are considered. First, all diseases identifiers are extracted from the indication, ChEMBL evidence and EuropePMC evidence json files. Disease identifiers that are not found in the MONDO ontology stored in DISEASE_ENTRY is looked up first by primary disease name, then disease synonyms. Alternatively, they can be searched by identifier against DISEASE_EXTDB and at last by matching 3^rd^ party identifiers between MONDO and EFO. If a match is identified, the EFO entry is added as an external identifier to a MONDO record in DISEASE_EXTDB. If for a given EFO record no match could be found, then each parent of that record will be matched following the same rules against a MONDO record in DISEASE_ENTRY table. Once identified, the EFO record will be added to DISEASE_ENTRY and its relationship to the parent record in MONDO will be made. This is to ensure completeness of disease description in the DISEASE_ENTRY table. Once done, a nested set representation is generated and stored in DISEASE_HIERARCHY. Next, drugs for which a drug type is provided are saved in DRUG_ENTRY table with their current approval/withdrawn state, max clinical phase, description and drug type. If a drug is a small molecule, it will be matched against SM_ENTRY and SM_SOURCE tables. Synonyms and trademark names are stored in DRUG_NAME.

ChEMBL evidence is then processed to associate a drug to its target and disease into DRUG_DISEASE table. For each triplet drug-disease-target, a lookup is performed against DRUG_ENTRY, DISEASE_ENTRY and GENE_SEQ respectively. For the latter, the corresponding NCBI Gene ID is retrieved. Thus, only triplets completely mapped to database records are stored in the DRUG_DISEASE table. Clinical trial identifiers are extracted from NTC, ATC, DailyMed and FDA URLs, and saved in CLINICAL_TRIAL table along with the clinical phase, status and study start date. The association between a clinical trial and a triplet drug-disease-target is realized thanks to the CLINICAL_TRIAL_DRUG table. All Open Targets indications, for which no targets are reported, are then processed. Thus, corresponding drug records are found by looking at either SM_NAME or DRUG_NAME tables, while corresponding disease records are found by searching by disease tag, name, synonyms or external identifiers via DISEASE_ENTRY, DISEASE_SYN and DISEASE_EXTDB tables. Novel pairs drug-disease are subsequently saved in DRUG_DISEASE table. New clinical trials are then stored in CLINICAL_TRIAL and its association with DRUG_DISEASE saved in CLINICAL_TRIAL_DRUG.

Gene to disease evidence extracted from literature is then processed using the EuropePMC evidence file provided by OpenTargets. Each triplet of gene, disease and publication is saved in PMID_DISEASE_GENE and the corresponding section and textual evidence extracted from the publication is stored in PMID_DISEASE_GENE_TXT

**Pubmed queries.**

Human genes, drugs, diseases and tissues are searched in Title/Abstract (TIAB), Text word (TW) or Mesh Term (MH) across all publications using PubMed E-Utilities^19^. Words less than 4 characters are automatically discarded. Common words that increase false positive are either removed or replaced. For Human genes, the following query is performed: “([GENE_NAME_LIST]) AND ((gene) OR (protein))”, where [GENE_NAME_LIST] is the primary symbol and all aliases. All publications for a given gene are stored in PUBLI_GN_MAP table.

**Clinvar:**

Clinical variant information is downloaded monthly from the ftp server of NCBI Clinvar in xml format. Each Clinvar set is stored in CLINV_ENTRY table, and their assertion are saved in CLINV_ASSERT. Observations, measures and traits are respectively stored in CLINV_OBSV, CLINV_MEASURE and CLINV_TRAIT. Most observations report publications, which allows the connection between PMID_ENTRY table and CLINV_OBSV table by recording it in CLINV_OBSVDT table. Additionally, the measure section provides information about the gene and the location of the variant. CLINV_MEAS_GNMAP table associate a specific CLINV_MEASURE to a gene in GN_ENTRY, whereas CLIVN_MEAS_LOC table connects a measure to a variant from VARIANT_CHANGE. The latter is only applied in the case of insertion, deletion and single nucleotide variant by looking at the alternative allele, the chromosome and the position in the chromosome.

**Clinical Trials:**

US Clinical trials are retrieved using an API call on clinicaltrial.gov website: <https://www.clinicaltrials.gov/api/v2/studies?format=json&pageSize=1000>. The NCT Identifier, start date, brief and official title, clinical phase and brief summary are extracted from the json data structure and saved in CLINICAL_TRIAL table. Next, each arm with its label, type and description are stored in CLINICAL_TRIAL_ARM, while each intervention with its type, name and description are recorded in CLINICAL_TRIAL_INTERVENTION. A mapping between interventions and arms are described in CLINICAL_TRIAL_ARM_INTERVENTION_MAP. Based on either OpenTargets, DrugBank or simple text matching, a drug is associated to an intervention via CLINICAL_TRIAL_INTERVENTION_MAP. The condition studied in the clinical trial is recorded in CLINICAL_TRIAL_CONDITION. If the condition matches a disease, either exactly by name, or by exact synonym or by a combination of the words, then the connection with DISEASE_ENTRY is made. Publication, clinical trial aliases and company are individually recorded in CLINICAL_TRIAL_PMID_MAP, CLINICAL_TRIAL_ALIAS, CLINICAL_TRIAL_COMPANY_MAP, respectively.

**Small molecule processing:**

Any molecule from any source will undergo the same process. First, the SMILES string is separated by its different molecules – separated by a dot – and the longest string will be considered the main chemical structure, while the others will be sorted first alphabetically and then concatenated together to form the set of counterions. Next, A SMILES file is generated by providing the full smiles and for the name: a combination of the identifier, InChi, InChi-KEY, counterions, and the smiles as the main molecular structure, each separated by a |. Next, the full SMILES will be standardized using Fileconv and preferred_smiles from LillyMol toolkit^20^. A flag (T/F) will be added to the name based on whether the standardization was successful for this molecule. Next, the standardized full molecule SMILES string will be used to generated the InChi and InChi-KEYs using RDKit^21^. Counterions and main molecular structures will then be extracted and stored in a separated file to be standardized using LillyMol. A similar success flag will be added for the main molecular standardization process. Therefore, each record will have a standardized full molecule SMILES string, standardized main molecule SMILES string, standardized counterion SMILES string, InChi and InChi-KEY. The standardized main molecule SMILES string will be checked against SM_MOLECULE table and added if it doesn’t exist. Similarly, the standardized counterion SMILES string will be checked against SM_COUNTERION table and added if it doesn’t exist. The full molecule SMILES string, InChi, InChi-KEY, and SM_COUNTERION, SM_MOLECULE identifiers are recorded in SM_ENTRY. At last, the molecule identifier is saved in SM_NAME with its connection to the source. This process ensures that each main molecule and counterion are uniquely defined once in SM_MOLECULE and SM_COUNTERION respectively, and their combination of is uniquely defined in SM_ENTRY. Once recorded, a process will list all SM_MOLECULE records with no assigned scaffold and saved as a SMILES string. Using LillyMol toolkit, the scaffold for each molecule will be computed and saved in SM_SCAFFOLD table. A Hash is generated using the combination of all 4 descriptors: INCHI, INCHI_KEY, standardized main molecule and counterion SMILES. This hash is then subsequently used to create a MOLECULAR_COMPONENT and MOLECULAR_ENTITY record.

**SwissLipids:**

Data from SwissLipids is retrieved from <https://www.swisslipids.org/api/downloadData>. Lipid ID, SMILES (pH7.3), InChi (pH7.3) and InChi key (pH7.3) fields are extracted and the small molecule process previously described is followed. In addition, alternative names provided in Name, Abbreviation*, Synonyms*, CHEBI, LIPID MAPS columns are saved in SM_SOURCE. At last, the connection between lipid molecules and publications as provided in the PMID field is saved in SM_PUBLI_MAP. All records from SwissLipids that contains a wildcard in their SMILES are separately stored in LIPID_ENTRY as they represent a lipid class. Thus, a nested set representation is generated for those lipid classes and saved in LIPID_HIERARCHY table. At last, the connection between the different lipids saved in SM_ENTRY and their lipid class is provided in LIPID_SM_MAP.

**SureChEMBL:**

A similar process is followed for SureChEMBL, after downloading the molecules from <https://ftp.ebi.ac.uk/pub/databases/chembl/SureChEMBL/data/>. In addition to the molecules, patent identifiers provided in SureChEMBL are saved in PATENT_ENTRY table. The connection between a patent and a molecule is made through SM_PATENT_MAP table in which the field (Description; Claim; Abstract; Title; Image; MOL attachment) as well as the frequency are saved.

**ChEMBL:**

First, molecules listed in ChEMBL are downloaded from <https://ftp.ebi.ac.uk/pub/databases/chembl/ChEMBLdb/latest/chemreps.txt.gz>. For each compound, the SMILES string, ChEMBL ID, standard InChI and InChI-Key are retrieved and undergo the small molecule standardization process. Next, all genetic targets are first processed by retrieving all component_sequences which are not protein and tested against TAXON, GENE_SEQ and TRANSCRIPT tables for taxonomic, gene and transcript identification, respectively and stored in ASSAY_GENETIC. Similarly, all component_sequences which are PROTEIN are then processed and searched against PROT_SEQ that defines a protein isoform and is subsequently saved in ASSAY_PROTEIN. Based on the type of target, one or multiple ASSAY_GENETIC or ASSAY_PROTEIN records are associated to an ASSAY_TARGET record, based on a similar information provided by ChEMBL. The assay type, confidence, tissue, target type are then processed and stored in ASSAY_TYPE, ASSAY_CONFIDENCE, ASSAY_TISSUE and ASSAY_TARGET_TYPE tables respectively. For ASSAY_TISSUE, a mapping is performed against ANATOMY_ENTRY, which stores the anatomy and tissue ontology. Variants are extracted from different sequences in ChEMBL tables and saved in ASSAY_VARIANT and ASSAY_VARIANT_POS tables. To do so, the provided protein isoform and positions are looked up against PROT_SEQ and PROT_SEQ_POS tables and their relationships – when found – are saved. Cell lines provided by ChEMBL are also extracted and processed by mapping the Cellausorus accession ID against CELL_ENTRY Table and the relationship stored in ASSAY_CELL. Once all those metadata are processed, assay records from ChEMBL tables are then retrieved and the ChEMBL metadata identifiers are converted to BioRels metadata identifiers and then stored in ASSAY_ENTRY table. Publications associated to an assay are then saved in ASSAY_PMID_MAP table. At last, each activity data point, without any filter, is saved using the ASSAY_ENTRY and MOLECULAR_ENTITY record in the ACTIVITY_ENTRY table.

**DrugBank:**

All small molecule structures follow the same standardization process so they can be recorded in SM_ENTRY and have a corresponding entry in MOLECULAR_ENTITY. Next, biotype categories are processed and saved as DRUG_TYPE records. The ATC classification as provided in DrugBank is recorded into 2 tables: The ATC Code and title are saved in ATC_ENTRY table, while the classification is generated via a nested set representation in ATC_HIERARCHY. A drug record in DRUG_ENTRY is defined by either a ChEMBL ID or DrugBank ID or both. Additionally, several flags are provided by DrugBank to specify whether the drug is approved, withdrawn, experimental, investigational, nutraceutical, illicit and approved for veterinary use. Names and synonyms are stored in DRUG_NAME. External identifiers are also recorded in DRUG_EXTDB. In addition, DrugBank provides different levels of textual description: simple, clinical and complete, each recorded in DRUG_DESCRIPTION table. The mapping between a drug and its type and ATC codes are provided via DRUG_TYPE_MAP and DRUG_ATC_MAP, respectively. At last, the association between a clinical trial intervention and a drug as provided by DrugBank is recorded in CLINICAL_TRIAL_INTERVENTION_DRUG_MAP.

**OMIM:**

First, each OMIM number is associated to a gene or allele using the association files genemap2.txt, allelicVariants.txt, respectively. In addition, OMIM identifiers are extracted from the disease ontology external identifiers. Then the omim.xml file is downloaded, and each entry is processed depending on whether it is a disease, gene or variant record and as such, will be saved in DISEASE_INFO, GENE_INFO and VARIANT_INFO tables, respectively.

**Gene Reviews & LiverTox:**

Gene Revies and LiverTox documents are processed as News. The text of each document is extracted from XML and saved into NEWS table. The association to a gene, disease or drug as provided by the data sources are stored in NEWS_GENE_MAP, NEWS_DISEASE_MAP, NEWS_DRUG_MAP tables respectively.

**PubMed Central (PMC):**

First, an annotation file is created listing all possible scientific terms that can be used to annotate PubMed Central documents. Each of the following annotation is filtered if it is part of a pre-defined list that we manually curated. All drug names and synonyms which are long then 1 character are extracted from DRUG_NAME and DRUG_ENTRY table. For names shorter than 4 characters, the suffix “ drug” is added. The remaining entries are then added to the annotation file using different case: as provided, lowercase or first letter in uppercase. For diseases, all disease tags, names are included as well as synonyms that are of type EXACT. Values will less than 4 characters are added the suffix “ disease”. All values are then added to the annotation file using different case: as provided, first letter uppercase. For small molecules, all names provided by ChEMBL are saved to the annotation file if they are not numeric, float, less than 3 characters. For cell lines, Cellausorus Accession, cell name and synonyms are added with the suffix “ cell” only if they are 4 characters or longer, not numeric or float. If the Wild Type suffix (/WT) is not present in the value, we add an additional record with /WT suffix. All Gene ontology and anatomy tags, names, synonyms of type EXACT are added to the annotation file if they are 4 characters or longer, not numeric or float. All Human Genes with their symbol are added to the annotation file. In addition, alternative versions of the symbol are provided: with the prefix “h” and/or with the suffix “ gene”. Only symbol and synonyms of more than 3 characters are considered. A similar logic is applied to mouse gene, but with the “m” prefix. Clinical trials name, alias and types, as well as company names are also saved in the annotation file.

To prepare the process, the open access file list is downloaded from PMC server. Only articles with a Creative Commons License of any type are considered and their archives downloaded. Next, the XML file is processed to extract and linearize the article into paragraphs with the additional images and tables. Each paragraph is further broken down into the individual lines where all annotations, HTML tags are temporary removed and special characters changed to alphabetical letters. The line is then tiled into a set of words, generating all possible combination of consecutive words. Each combination is then tested against the annotation file for a possible match against a gene, disease, drug, compound, gene ontology, cell line or tissue. If a match is found, we then check if an acronym is provided in parenthesis after the matched words. If an acronym - defined as a word in brackets - is found but not associated to any annotation, then that acronym is excluded from future potential annotations. If so, the acronym is then check for a match in the rest of the article. A match is then defined by the paragraph number, the line number, the position of the starting word and the number of words for the match. To record the data in the database, each article is defined in PMC_ENTRY table. Each raw paragraph is then saved in PMC_FULLTEXT and the paragraph order defined by the offset from the beginning of the article. If multiple paragraphs are part of the same section, a group ID is provided to uniquely group them together. Next, each annotation match is recorded with its positioning information into PMC_FULLTEXT_CLINICAL_MAP, PMC_FULLTEXT_CELL_MAP, PMC_FULLTEXT_COMPANY_MAP, PMC_FULLTEXT_ANATOMY_MAP, PMC_FULLTEXT_DRUG_MAP, PMC_FULLTEXT_DISEASE_MAP, PMC_FULLTEXT_SM_MAP, PMC_FULLTEXT_GENE_MAP, PMC_FULLTEXT_GO_MAP, PMC_FULLTEXT_PUB_MAP for clinical trial, cell line, company, anatomy/tissue, drug, disease, small molecule, gene, gene ontology and publication citations, respectively.

**Identification of RNA Editable sites:**

We selected all NCBI genes associated to Homo Sapiens (TaxID:9606) and that are defined as protein-coding. For each gene, we retrieved their strand information as well as all transcripts that are tagged as mRNA or protein-coding. For each transcript, we look at individual position to evaluate if an adenosine is reported. To do so, 3 rules are applied for each individual transcript position:

- The reported transcript nucleotide is A
- If the gene is on the negative strand and:
  - the corresponding chromosome nucleotide is T
  - a reported single nucleotide variant is reported as T
- If the gene is on the positive strand:
  - the corresponding chromosome nucleotide is A
  - a reported single nucleotide variant is reported as A

This allows to take into consideration low frequent variant as well as potential missing nucleotides due to modification of the transcript sequence by the RefSeq team. All corresponding chromosome sequence position are saved in a table called RNA_EDIT_ENTRY. All corresponding transcript position that reports an adenosine according to the rules above are stored in RNA_EDIT_TRANSCRIPT.

Next, for each RNA Editable site, we use mRNA to protein translation to verify whether the nucleotide change will trigger a protein-recoding. To do so, we retrieve for each RNA Editable site the corresponding transcript position and the corresponding codon in the mRNA/protein translation, noting the position within that codon. The nucleotide in that specific codon position will first be changed to A – irrelevant to what it currently is - and the corresponding amino-acid is deducted from the Standard translation table. The pair codon and amino-acid is called current editable triplet (CET). Next, the nucleotide in that specific codon position will first be changed to G and the corresponding amino-acid is deducted from the Standard translation table. The edited pair codon and amino-acid is called edited editable triplet (EET). Thus, we consider it a potential protein-recoding editable event if the CET and EET amino-acid is different and record it in the RNA_EDIT_PROTEIN.

1 NCBI. *NCBI Taxonomy FTP Server*, <<ftp://ftp.ncbi.nih.gov/pub/taxonomy/taxdump.tar.gz>> (

2 Scott, F. The Taxonomy Project. *McEntyre J, Ostell J, editors. The NCBI Handbook [Internet]* (2002).

3 NCBI. NCBI Gene ftp server.

4 Eyre, T. A., Wright, M. W., Lush, M. J. & Bruford, E. A. HCOP: a searchable database of human orthology predictions. *Brief Bioinform* **8**, 2-5 (2007). <https://doi.org/10.1093/bib/bbl030>

5 Braschi, B. *et al.* Genenames.org: the HGNC and VGNC resources in 2019. *Nucleic Acids Res* **47**, D786-D792 (2019). <https://doi.org/10.1093/nar/gky930>

6 Jassal, B. *et al.* The reactome pathway knowledgebase. *Nucleic Acids Res* **48**, D498-d503 (2020). <https://doi.org/10.1093/nar/gkz1031>

7 Ashburner, M. *et al.* Gene ontology: tool for the unification of biology. The Gene Ontology Consortium. *Nat Genet* **25**, 25-29 (2000). <https://doi.org/10.1038/75556>

8 The Gene Ontology Consortium. The Gene Ontology Resource: 20 years and still GOing strong. *Nucleic Acids Res* **47**, D330-D338 (2018). <https://doi.org/10.1093/nar/gky1055>

9 *Journal list from PubMed*, <<https://ftp.ncbi.nlm.nih.gov/pubmed/J_Entrez.txt>> (

10 Mitchell, A. L. *et al.* InterPro in 2019: improving coverage, classification and access to protein sequence annotations. *Nucleic Acids Res* **47**, D351-D360 (2018). <https://doi.org/10.1093/nar/gky1100>

11 Altschul, S. F., Gish, W., Miller, W., Myers, E. W. & Lipman, D. J. Basic local alignment search tool. *J Mol Biol* **215**, 403-410 (1990). <https://doi.org/10.1016/s0022-2836(05)80360-2>

12 Smith, T. F. & Waterman, M. S. Identification of common molecular subsequences. *Journal of Molecular Biology* **147**, 195-197 (1981). <https://doi.org/https://doi.org/10.1016/0022-2836(81)90087-5>

13 Henikoff, S. & Henikoff, J. G. Amino acid substitution matrices from protein blocks. *Proceedings of the National Academy of Sciences* **89**, 10915-10919 (1992). <https://doi.org/10.1073/pnas.89.22.10915>

14 *dbSNP Frequency studies*, <<https://ftp.ncbi.nlm.nih.gov/snp/latest_release/JSON/frequency_studies.json>> (

15 *dbSNP ALFA build summary 2020*, <<https://www.ncbi.nlm.nih.gov/snp/docs/gsr/alfa/ALFA_20200227123210/>> (

16 NCBI. *dbSNP FTP server*, <<https://ftp.ncbi.nlm.nih.gov/snp/latest_release/JSON/>> (

17 Carvalho-Silva, D. *et al.* Open Targets Platform: new developments and updates two years on. *Nucleic Acids Res* **47**, D1056-D1065 (2018). <https://doi.org/10.1093/nar/gky1133>

18 Open Target FTP server.

19 NCBI. (2010).

20 ELaC, C. C. a. C. G. *LillyMol Public Code*, <<https://github.com/EliLillyCo/LillyMol>> (2018).

21 Landrum, G. *et al.* rdkit/rdkit: 2024_03_1 (Q1 2024) Release. (2024). <https://doi.org/10.5281/zenodo.11102446>
