# Supplementary figures and images for: BioRels’ data infrastructure: a scientific schema and exchange standard to transform and enhance biological data sciences
Source: Nucleic Acids Res. 2025 Apr 4;53(6):gkaf254. doi: 10.1093/nar/gkaf254 (PMC11969666; doi:10.1093/nar/gkaf254)

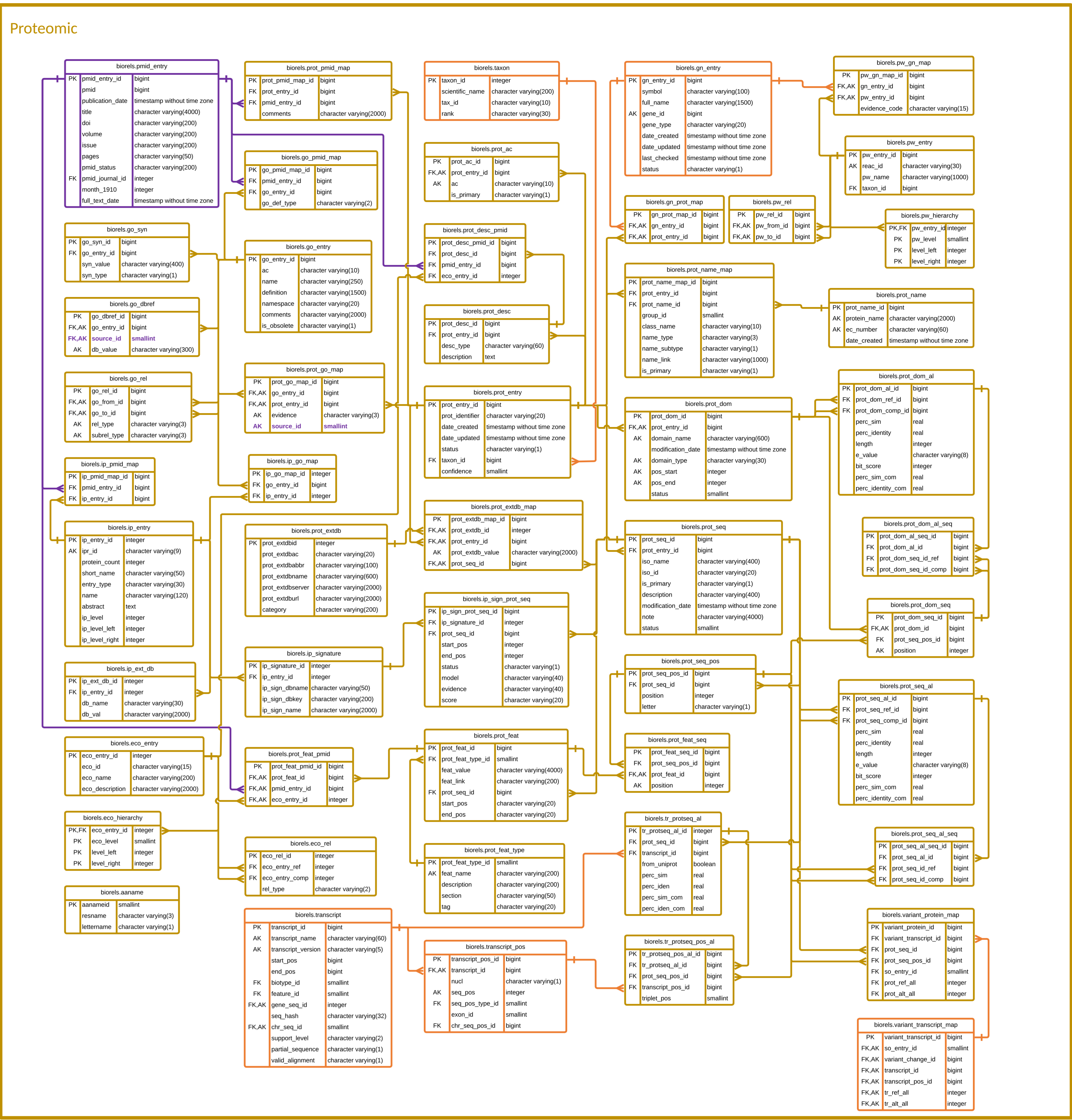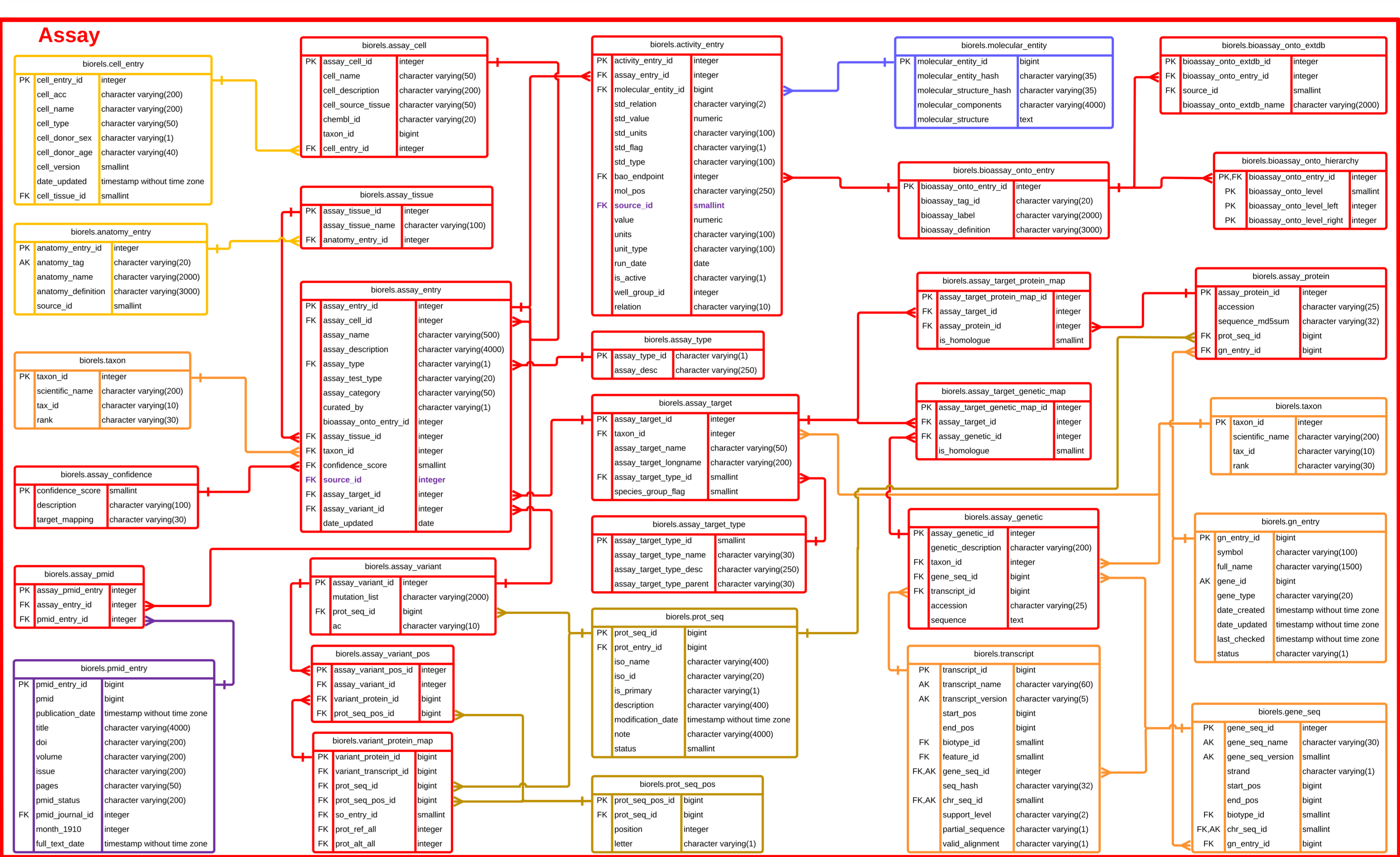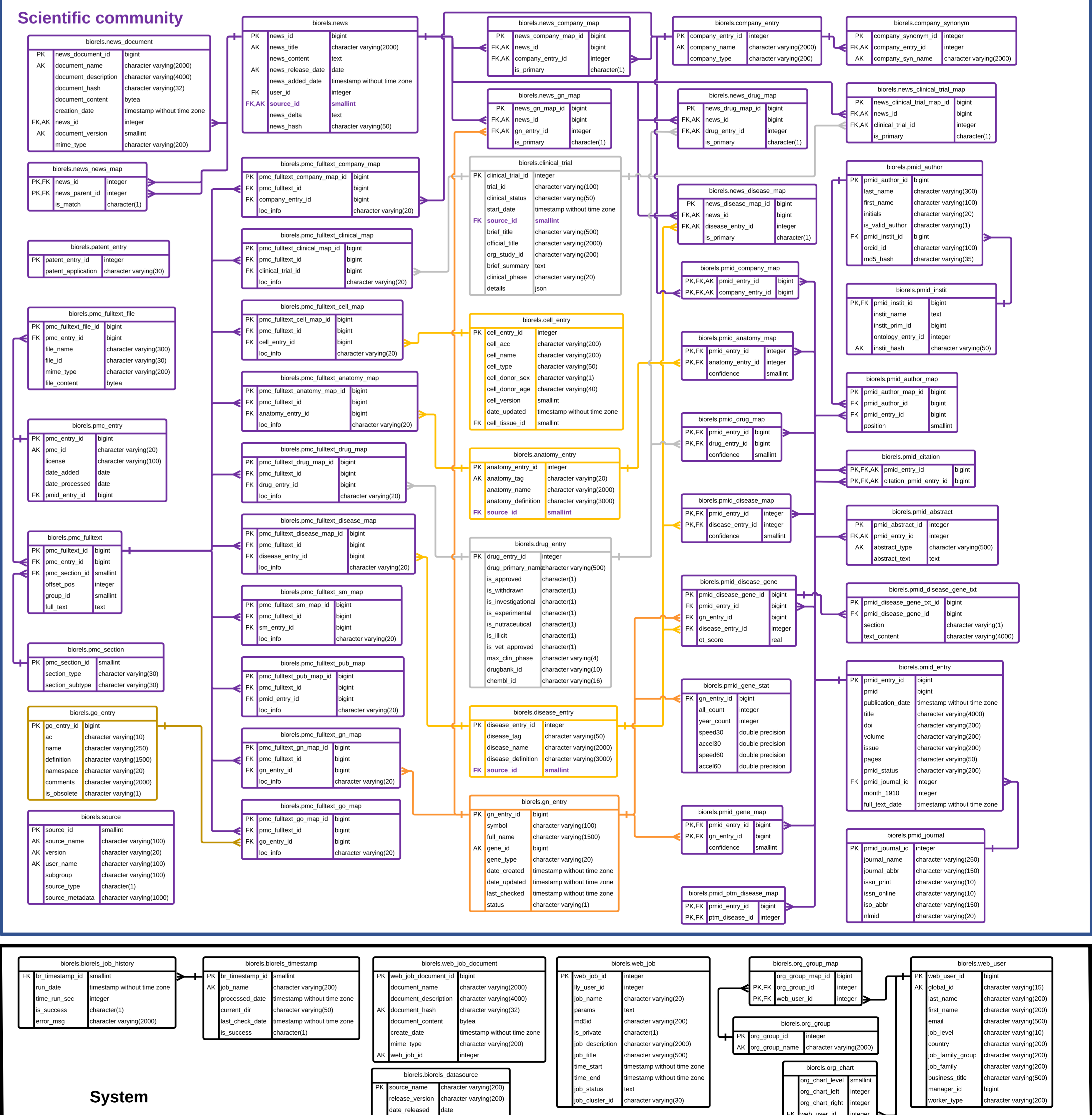

Supplement: gkaf254_Supplemental_Files [file gkaf254_supplemental_files.zip › Supplementary Figure 1.pdf]
